# High Pressure X-Ray Crystallography With the Diamond Cell at NIST/NBS

**DOI:** 10.6028/jres.106.045

**Published:** 2001-12-01

**Authors:** Gasper J. Piermarini

**Affiliations:** National Institute of Standards and Technology, Gaithersburg, MD 20899-8522

**Keywords:** crystallography, diamond anvil cell (DAC), high pressure, hydrostaticity, polycrystalline, ruby pressure measurement, single crystal

## Abstract

Scientists in the Crystallography Section at NIST/NBS made several outstanding contributions which greatly promoted the development and advancement of high pressure x-ray crystallography during the second-half of the 20th century. These milestone achievements or “firsts” included: (1) the invention of the lever-arm type diamond anvil cell (DAC) in 1958; (2) the development of DAC technology for powder x-ray diffraction at high pressure in 1960; (3) the introduction of DAC methodology for single crystal x-ray diffraction at high pressure in 1964; (4) the invention of the optical fluorescence ruby method of pressure measurement in 1971; and (5) the discovery of hydrostatic pressure-transmitting media useful to unprecedented pressures for that time. These achievements provided the spark that ignited the explosion of activity in high pressure research that occurred in laboratories throughout the world during the latter part of the 20th century. It is still going on, unabated, today. An estimated 5000 DACs were built during the last 40 years.

## 1. Introduction

Two closely related world-class scientific and technological achievements occurred in the field of high pressure research at the National Bureau of Standards (NBS), now the National Institute of Standards and Technology (NIST), during the second-half of the 20th Century: (1) the invention of the lever-arm type diamond anvil high pressure cell in 1958, and (2) the invention of the ruby fluorescence method of pressure measurement in 1971. These two developments, probably more than any others, stimulated the profound advancement of high pressure research that evolved during the last 30 years both at NBS and other scientific laboratories throughout the world.

The work leading to these accomplishments took place in the Constitution and Microstructure Section (CMS), renamed the Crystallography Section (CS) in 1964. Soon after the invention of the lever-arm type diamond anvil cell (DAC), a high pressure research program utilizing the DAC was initiated in CMS and was active for almost 37 years. During those years, milestones or “firsts” were made in several areas including: the development of DAC technology for obtaining x-ray powder diffraction data at high pressure; use of a metal gasket to encapsulate liquids and other materials under pressure between the anvils of a DAC; the development of the methodology for determining crystal structures at high pressure in the DAC from single crystal x-ray diffraction intensity data (first application: growth and structure of a single crystal of ice VI); the invention of the ruby fluorescence technique for pressure measurement; the generation of ultrahigh pressures through improvements in the design of the original NBS DAC; the measurement of hydrostatic limits in pressure-transmitting media utilizing the ruby method; the application of the DAC to Raman spectroscopy; and the measurement of the viscosity of liquids at high pressures in the DAC by the classical Stokes method.

In the spirit of the NIST Centenary and this Special Issue to honor it, the content of this article is limited to a description of the role CMS and CS played in influencing the development and advancement of high pressure x-ray crystallography during the early years of its infancy, from 1959 to 1975. The author acknowledges the many outstanding accomplishments with the DAC by other laboratories in the United States and abroad, including the attainment of megabar pressures and the application of the DAC to many scientific measurement techniques, too numerous to mention here. Certainly, all provided additional impetus for the further advancement of high pressure research with the DAC. To maintain focus within the framework and scope of this Special Issue, this report emphasizes the seminal nature of the work accomplished at NBS in the years immediately following the invention of the DAC when work with it in other laboratories was just beginning.

## 2. The Invention of the Diamond Anvil Cell (DAC)

The invention of the DAC took place in CMS (then headed by H. F. McMurdie) of the Mineral Products Division (I. C. Schoonover, Chief) in 1958. The NBS laboratories were then located in Washington, DC. From the beginning, the DAC was designed as a lever-arm device with a 180° optical transmission path, and was conceived for use in infrared (ir) spectroscopy at high pressure. Indeed, its first application was to obtain ir absorption spectra for the first time on powders squeezed between two opposed diamond anvils [1].

**Fig. 1:**
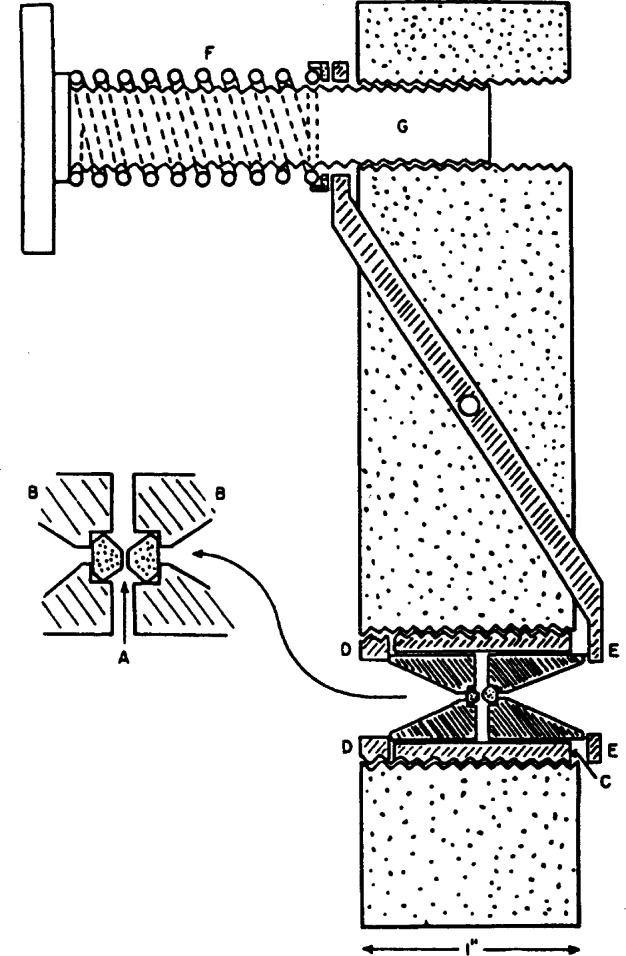
Schematic cross section diagram of the original diamond “squeezer” for infrared transmission studies to 3 GPa. The insert magnifies the opposed diamond anvil configuration to show greater detail.

A schematic cross section diagram of the original design of the instrument is shown in Fig. 1. The device contains two gem-cut type II diamonds, each about 1/3 carat in size, comprising the “squeezer” anvils (inset). The culet of each diamond contains a small flat, parallel to the table. The powdered specimen is placed between these opposed flats, having an area of about 0.13 mm^2^. Each diamond, A, is seated on its tabular face which rests in a close-fitting recess in a stainless steel piston, B. Each piston has a conical hole to permit acceptance of the maximum flux from a convergent cone of radiation passing through both pistons, the diamonds, and the powdered specimen pressed between them. The pistons are free to slide in a cylindrical bearing, C, that screws into a large block of steel carrying the pressure-generating mechanism. The entablature piston is supported by a threaded thrust-bearing ring, D. The presser-plate. E, connected to a lever, pivoted in the block and activated by a calibrated spring, F, bearing against the upper end of the lever, pushes against the other piston. The spring is compressed by the manually operated screw, G. Because of the small sample size, the device had to be used with a set of beam condensing parabolic mirrors in order to obtain sufficient ir energy throughput.

Some of the very first materials studied were NaNO_3_, KNO_3_, AgNO_3_, ferrocene, ice, and CaCO_3_. These were pioneering experiments because they demonstrated for the first time that pressure-dependent shifts and changes in intensity of infrared absorption bands could be measured. The absorbance of most bands was observed to weaken with increasing pressure. In the case of calcite (CaCO3), the pressure-shifts were related quantitatively to a decrease in the C-O bond length, a result compatible with the reported compressibility of calcite perpendicular to the trigonal axis. In addition, large changes in spectra resulting from pressure-induced phase transformations were observed. Results such as these, opened up a whole new area of study with the DAC for infrared spectroscopy.

The invention was not the result of formally planned research. Rather, it was the result of an evolving interaction among three NBS scientists, C. E. Weir, A. Van Valkenburg and E. N. Bunting, and a consultant from the University of Maryland, E. R. Lippincott—four scientists with different interests, different research activities and different backgrounds, who interacted on a professional level and recognized an opportunity to make a significant contribution to scientific research. Together, they produced this remarkable instrument we call the diamond anvil cell.

In the early stages of the DAC’s development, funds to support it directly were not available. However, NBS scientists had the freedom to pursue nonfunded personal interest research, provided it did not interfere with official duties and the work was related to goals of their research project. NIST Scientists still have the freedom to do this today. In the very early stages of the DAC developmental work, these four scientists used their own initiative and ingenuity to get things accomplished. Thus, the pressure cell was handmade by Weir, who fabricated it utilizing only equipment that was available in his laboratory, e.g., a lathe, drill press, hack saw, soldering gun, threading tools, files, and a high speed grinding wheel to polish down the culets of the brilliant-cut gem diamonds to form anvil faces. The gem diamonds were obtained by Van Valkenburg from the General Services Administration (GSA), the custodian of diamonds confiscated from smugglers by customs agents. At that time, GSA contraband diamonds were available to other government agencies at no cost, if their use in supporting government programs could be justified. Without these gratis gem diamonds, the DAC probably would not have been perfected as rapidly as it was, because, during the early stages of its development, many diamond anvils were destroyed in the testing process. To replace them commercially would have been prohibitively expensive, an expenditure not easily justified.

**Fig. 2:**
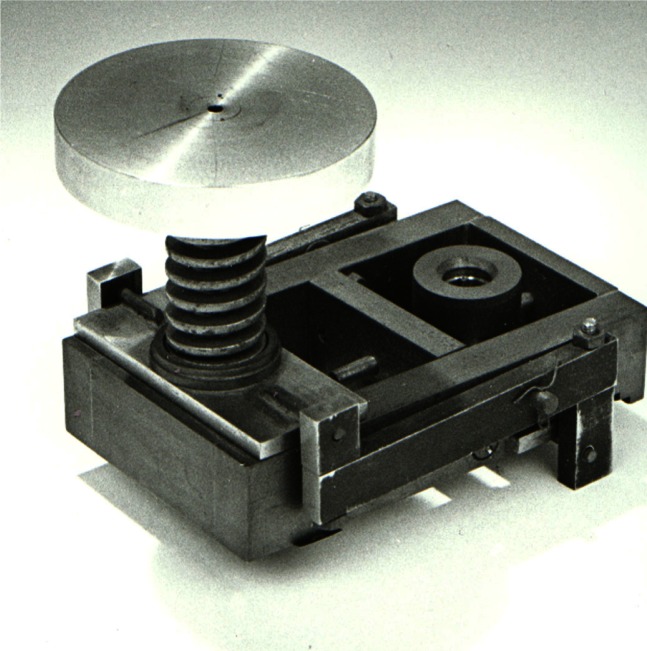
The original diamond anvil pressure cell, now on display in the NIST Gaithersburg Museum. The unrefined instrument was handmade by C. E. Weir at NBS in 1957–58.

A photograph of the original NBS instrument, small enough to fit in the palm of one’s hand, is shown in Fig. 2. Close inspection confirms that the device was crudely made utilizing simple machine tools and fabrication procedures. Yet, the basic design shown here is the prototype for most DACs with 180° optical transmission even to the present day. It was truly an ingenious design, dictated by the limited fabrication equipment available to them. Indeed, what were considered to be great hindrances to its fabrication, turned out in the end to be a great boon. Because of its miniature size and simplicity in design and use, it was quickly adopted by spectroscopists worldwide. Not only was it used for its intended purpose, i.e., to study the effects of pressure on the infrared spectra of materials subjected to high pressures, but it also had a completely unexpected application in crime laboratories as a microsample holder for traces of evidence found at the scene of a crime. It is still used today for this purpose.

In what appears to be a case of almost simultaneous discovery, because neither group was aware of the other’s work, a similar device was built by J. C. Jamieson and coworkers at the University of Chicago for x-ray powder diffraction studies [2]. Their device, described in the literature 3 months after a detailed description of the NBS instrument had been published, also made use of diamonds in a Bridgman opposed anvil arrangement, but the fundamental difference between the two instruments was that the University of Chicago cell did not have 180° optical access and required a hydraulic press to apply load to the anvils. It was designed specifically for use on a horizontal x-ray diffractometer. The instrument did not enjoy widespread use because it lacked portability, ease of use, and adaptability to other scientific measurement techniques.

## 3. Crystallographic Studies at High Pressures

Crystallographic studies at high pressure with the DAC began in 1960, just 2 years after it was first used in the laboratory for ir studies. The first advance was made in x-ray powder diffraction. Then, after the development of the gasket technique which permitted encapsulation of liquids and crystals in liquids in the DAC, advances were made in single crystal x-ray diffraction. Both of these applications utilized film methods which were in prevalent use at that time. Also the DAC was easily adapted to the instrumentation available for film techniques. The powder and single crystal advances made at NBS were truly significant contributions to high pressure x-ray crystallography. Both demonstrated the power of the DAC in acquiring crystallographic information at high pressures. They served as the foundation for later work in both areas as advanced x-ray sources and detection technology came into being.

### 3.1 Powder Diffraction

**Fig. 3:**
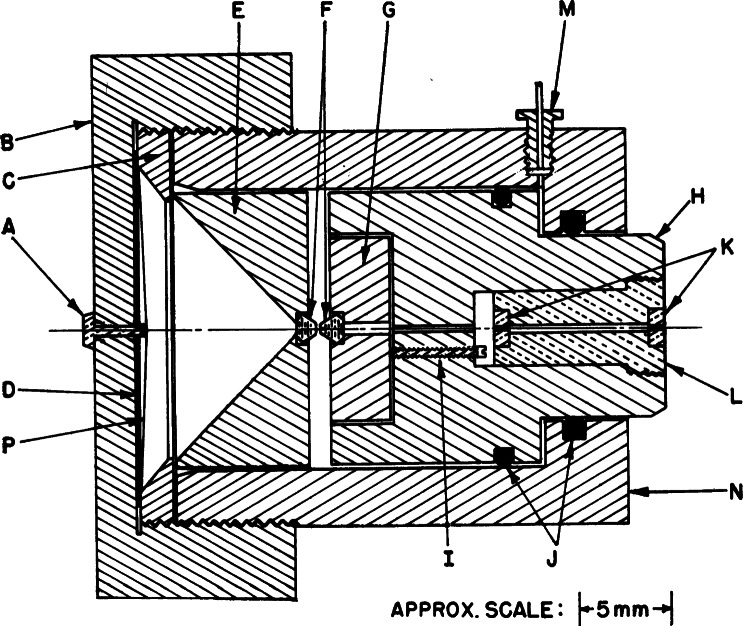
Schematic cross section diagram of the hydraulically-loaded miniature high pressure x-ray powder camera designed by C. E. Weir at NBS in 1960.

In 1960, Weir had redesigned the lever-arm cell into a miniature hydraulically-loaded device for x-ray powder diffraction measurements. To demonstrate the new instruments capability, he spent the summer of that year in Prof. Leason H. Adams’ x-ray laboratory at the University of California in Los Angeles, where he succeeded in obtaining transmission powder patterns on a circular flat film. A schematic cross section diagram of his design of the x-ray powder camera for high pressure studies is shown in Fig. 3. The unit contains a hardened steel entablature (E) and a piston (H) actuated by hydraulic pressure generated by an external hand pump and introduced through a fitting (M). The hydraulic liquid, confined to the small volume between the rubber O-rings (J), exerts an axial load on the shoulder of the piston (H). The entablature (E) is supported by the thrust washer (C) and the screw-cap (B). The entablature and the piston are both fitted with brilliant-cut gem-quality diamonds (F), approximately 0.15 carats. The diamonds are seated on their table faces in small close-fitting recesses. The piston diamond is seated in a small cylindrical steel insert (G) which can be tilted by three steel screws 120° apart (I) to permit parallel alignment of the opposed diamond faces. The x-ray beam enters through two collimating pinholes (K), 0.355 mm in diameter. Zirconium filtered molybdenum radiation was used which traverses both diamonds and the film of powder compressed between the anvils. Diffraction rings from the powdered specimen diverge through the diamond in the entablature and are recorded on the film (D), placed inside the cap and held securely between cap and thrust washer. The film is covered with a light protective paper (P) and a small beam stop (A) secures the film at the center. The entablature is machined out to form a conical surface with a 45° semivertex angle to permit a wide divergence of the diffraction rings. The sample-to-film distance was approximately 10 mm. Under the experimental conditions used here diffraction rings with 2*θ* > 20° could not be recorded on the film.

On his return to NBS, Weir continued to work with the new powder camera, further exploring its potential uses. Shortly thereafter, I began a collaboration with him in that effort, and introduced several improvements in the design of the instrument which included: (1) a reduction in the x-ray beam divergence and collimation diameter to 0.15 mm to minimize the effect of pressure gradients in the pressed sample; (2) an increase in the sample-to-film distance to include a range between 30 mm and 75 mm for improved accuracy in the measurement of diffraction ring diameters; (3) a corresponding increase in film-cassette diameter to 75 mm; (4) a modified entablature containing a wedge cutout to permit the recording of diffraction rings on film to 2*θ* ≈ 35°. The circular film cassette incorporated a split-field baffle permitting simultaneous exposure only of opposite quadrants of the film. A simple 45° rotation of the baffle permitted a second independent exposure on the same film. Because the thermal expansion of the hydraulic fluid was sensitive to small changes in the ambient room temperature, the applied load on the anvils (sample pressure) varied significantly during the course of an experiment, often significantly broadening the width of the diffraction rings. To minimize this undesired temperature effect, water at constant temperature was circulated through a Cu tube coiled around the base of the instrument. These combined improvements produced a relatively large diameter, narrow width, well defined diffraction ring on film, ultimately yielding a more accurate measurement of its diameter. The first report describing the x-ray powder camera along with results obtained with it, was published in 1962 [3]. The instrument was used initially to study the effect of pressure on AgI, Bi, Tl, and KI, but later work included most of the alkali halides and four rare earth elements, La, Ce, Pr, and Nd. [4–6]. Many of these materials were known to exhibit pressure-induced phase transitions, while others were thought to be good candidates for high pressure polymorphism. In many cases the crystallography of the high pressure phases was unknown and was the primary reason for studying them.

**Fig. 4:**
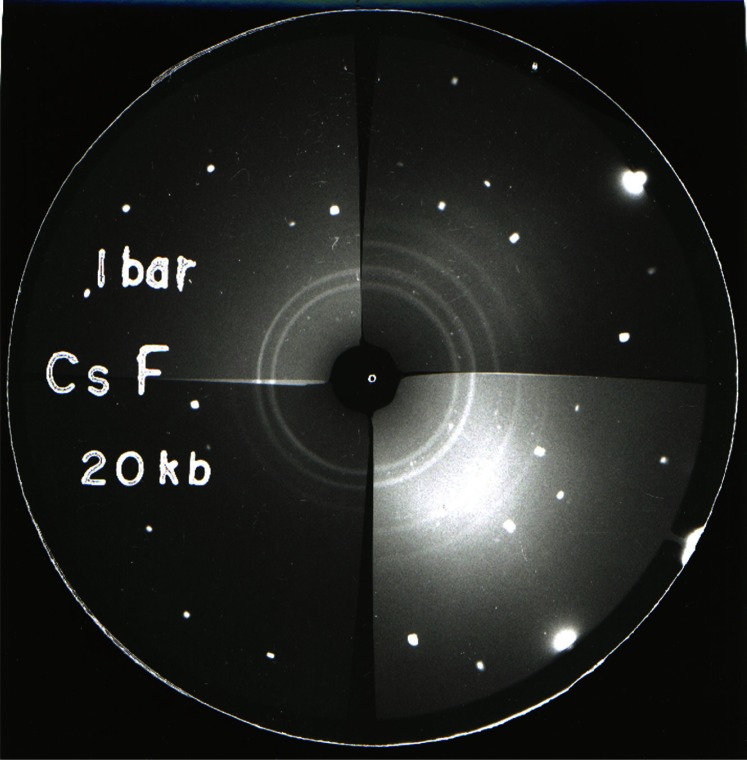
Matched pair (opposed quadrants) of x-ray powder patterns of CsF taken at ambient pressure and 2.0 GPa (20 kbar) illustrating a pressure-induced phase transformation. The stable NaCl-type structure converts to a CsCl-type structure at approximately 2 GPa.

**Fig. 5:**
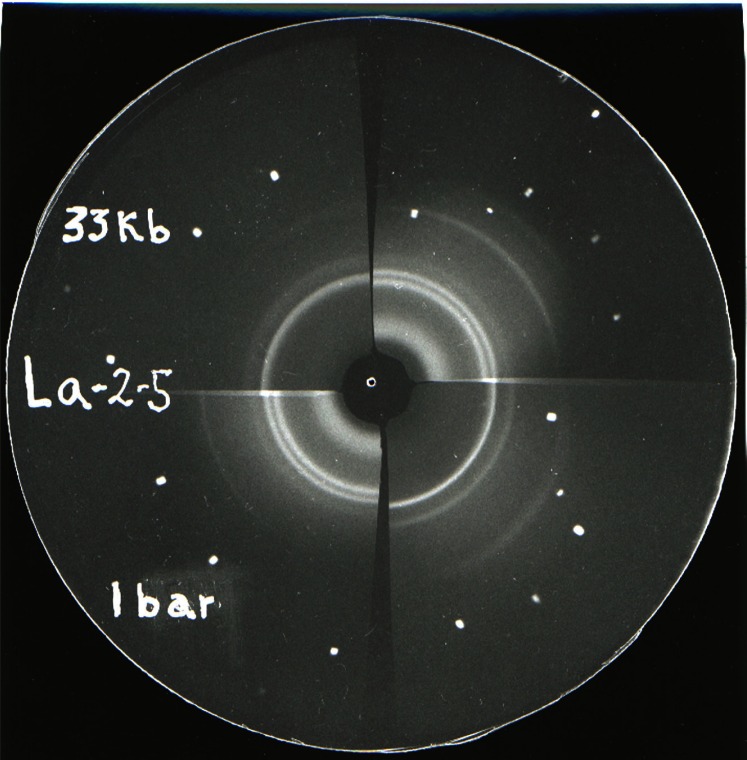
Matched pair (opposed quadrants) of x-ray powder patterns of the rare-earth element, La, taken at ambient pressure and 3.3 GPa (33 kbar). The hexagonal structure, stable at ambient pressure, transforms to a cubic-closed-packed structure at about 3.3 GPa.

Typical examples of diffraction patterns recorded on film are shown in Figs. 4 and 5 for CsF and La, respectively. Both illustrate a pressure-induced phase transition. Cesium fluoride normally has a NaCl-type structure and was shown to convert to a CsCl-type structure at approximately 2.0 GPa. Previous reports on the alkali halides indicated that pressure-induced polymorphism was absent in the fluorides, and that the fluorides were anomalous in this respect. However, in this work similar transformations were found also for the fluorides of potassium and rubidium. The transitions were confirmed by visual microscopic examination of powders pressed in a DAC. In La metal, the ambient pressure hexagonal phase transforms to a cubic-close-packed structure at about 3.3 GPa. The four elements studied, La, Ce, Pr, and Nd, have similar electronic configurations. Except for Nd, reported compression and electrical resistance measurements indicated that pressure-induced phase transitions occurred in these metals, but the crystallography of the high pressure phases was unknown. The results of our x-ray studies showed that La, Pr, and Nd transformed from the normal hexagonal La-type structure to a face-centered cubic (fcc) structure. Cerium metal, however, behaved quite differently from the other three. It was found to transform from a fcc structure to a “collapsed” fcc structure. at 0.75 GPa. The transition is thought to involve the promotion of a 4*f* electron to the 5*d* band.

**Fig. 6:**
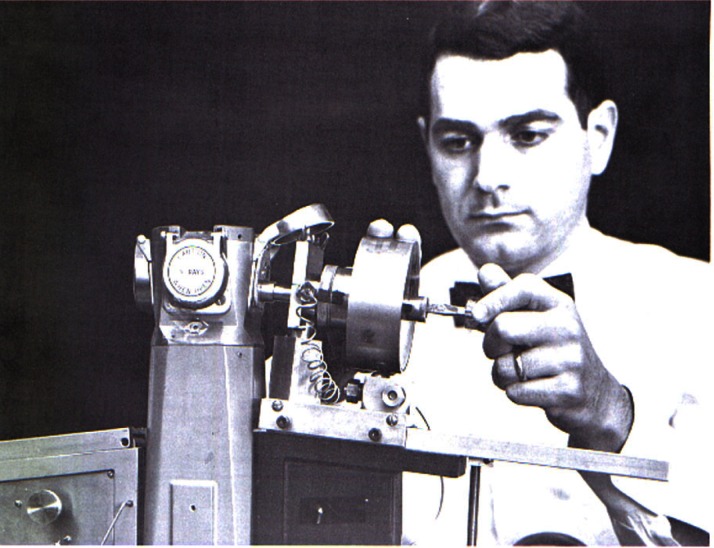
A photograph, taken at NBS in 1964, of the hydraulically-loaded high pressure x-ray powder camera mounted on the table of an x-ray unit. This is the improved version of the design shown in Fig. 3. The film cassette is being adjusted by G. J. Piermarini.

This work was very important to crystallography because not only did it demonstrate that x-ray powder diffraction patterns could be obtained routinely from a DAC, but it also showed some interesting effects of pressure on materials, e. g., compressibility and pressure-induced phase transformations. High-pressure polymorphism in CsF, RbF, KF, and Nd was shown for the first time. The high pressure forms of these alkali fluorides all were shown to be simple cubic phases, while the high pressure modifications of the rare-earth metals, La, Ce, Pr, and Nd, exhibited a face-centered cubic structure. The unit cell dimensions for these rare-earth metals were reported for the first time. A photograph of the refined version of the high pressure x-ray powder diffraction camera is shown mounted on the table of an x-ray unit in Fig. 6.

### 3.2 Gasket Technique

**Fig. 7:**
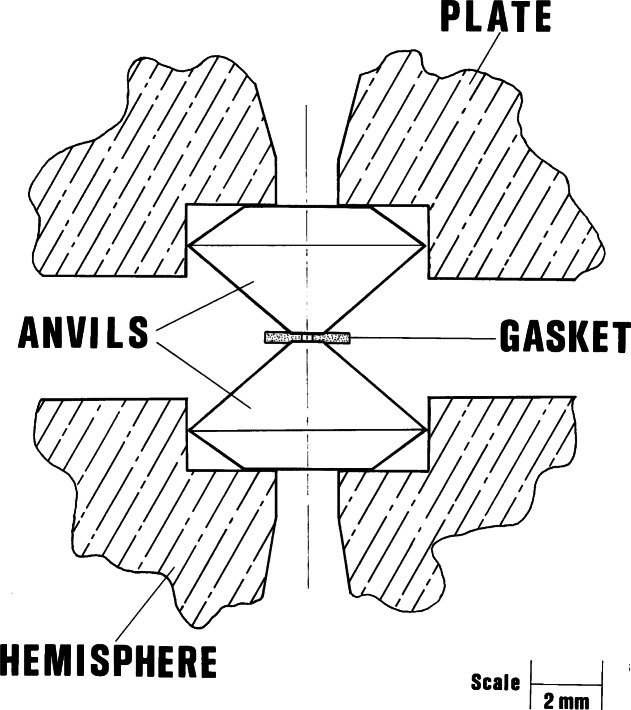
Schematic cross section diagram of the opposed diamond anvil configuration showing the gasket, a thin metal sheet (usually Inconel X750) with a small hole in it, placed between the anvil faces. The hole, containing the sample, is precisely centered over the anvil faces to prevent blow-out as the anvils are squeezed together to deform the gasket and decrease the sample volume.

**Fig. 8:**
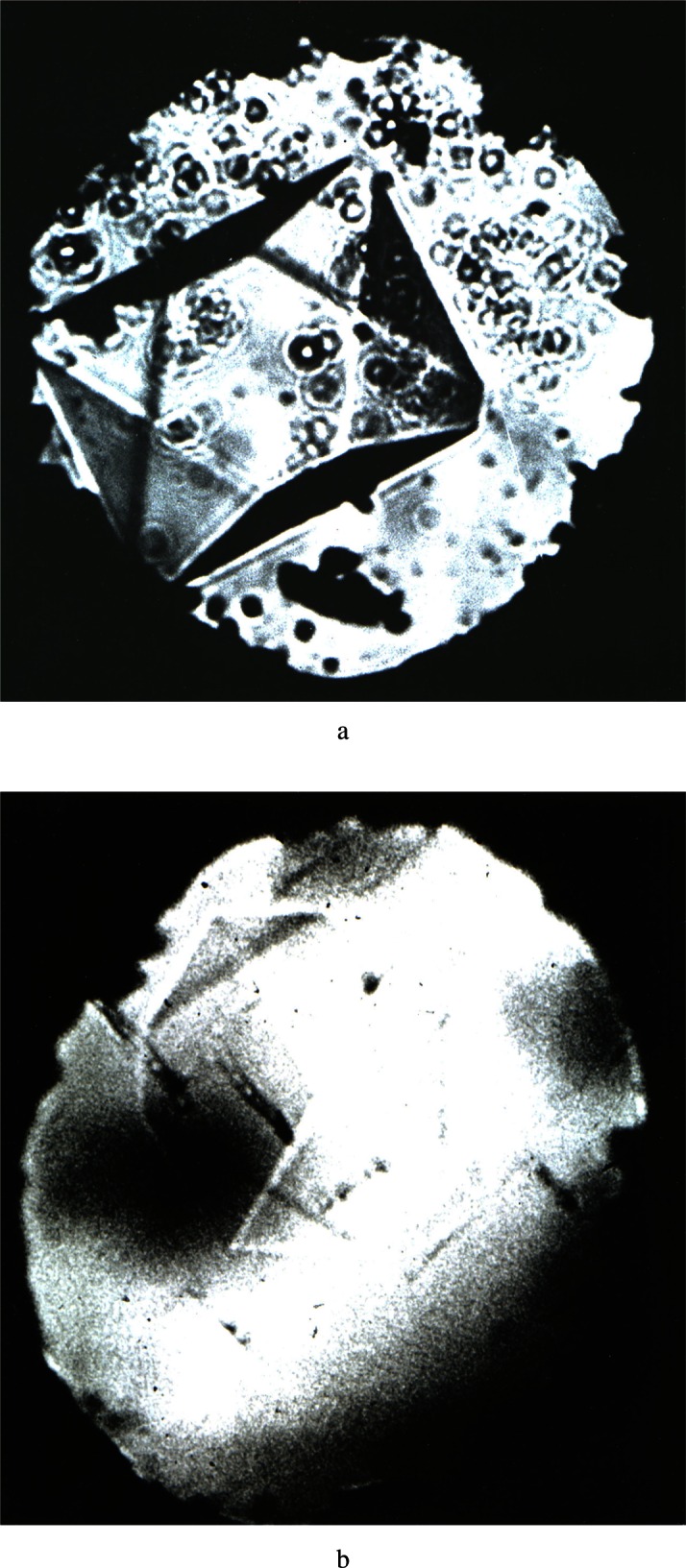
(a) A single crystal of ice VI in equilibrium with liquid at room temperature and about 0.96 GPa (9.6 kbar). (b) A single crystal of carbon tetrachloride (phase I) is shown in equilibrium with its liquid at room temperature and a pressure of about 0.13 GPa (1.3 kbar). The rhombohedral crystal exhibits a trigonal axis perpendicular to the plane of the diamond window.

In 1962, Van Valkenburg developed a gasket method to confine liquids in the DAC by placing a thin metal sheet with a small hole containing the liquid between the anvil faces as shown in Fig. 7 [7]. Initially, the technique was used to study the freezing behavior of many liquids such as water, methanol, ethanol, carbon tetrachloride, benzene and similar liquids by the application of pressure at room temperature (RT). Typical examples of this behavior as viewed through the diamond window of the pressure cell with the aid of a polarizing microscope are shown in Figs. 8a and 8b for single crystals of ice VI and carbon tetrachloride, respectively. The gasket technique was quickly extended to powders, mixtures of liquid and powder and liquid with single crystals of another material. As a result of these developments, one of the first applications of the gasket technique was in x-ray powder diffraction experiments with the hydraulic DAC in use at that time. Powders were confined with chemically inert liquids in a gasketed cell to provide a hydrostatic environment for the sample under pressure. Introduction of this technique resulted in improved powder diffraction patterns owing to the absence of stress inhomogeneity in the sample. To eliminate diffraction interference from the metal gasket, W collimator tubes (0.2 mm ID) with internal diameters much less than the gasket diameter were used. This procedure resulted in fine-quality powder diffraction patterns out to 2*θ* ≈ 35°.

### 3.3 Single Crystal Diffraction

The gasket technique was very important to crystallography, because, soon after we used it with chemically inert liquids to provide a hydrostatic pressure-transmitting environment for x-ray powder diffraction, we recognized almost immediately its potential application to single crystal x-ray diffraction. Chronologically, the high pressure single crystal diffraction technique evolved stepwise as potential applications of the DAC became more apparent with use. Within two years of its inception, the original DAC for infrared spectroscopy evolved, as we saw, into the miniature hydraulically actuated device for x-ray powder diffraction. Almost simultaneously, the gasket technique for encapsulating liquids, coupled with optical polarizing microscopy for viewing the sample, was introduced. Shortly thereafter, optical microscopy observations with polarized light in a gasketed cell revealed that defect-free single crystals could be grown readily from many liquids, e. g., water, methanol, ethanol, benzene, carbon tetrachloride, etc., at room temperature (RT) by the application of pressure. Moreover, the crystals could be retained in the cell at pressure indefinitely. These early discoveries with the gasket technique were important because they encouraged Stanley Block (who became chief of the Crystallography Section in 1965), Gasper Piermarini, and Charles Weir to investigate the possibility of studying single crystals at high pressure by an x-ray diffraction technique. In 1964, all high pressure x-ray studies involved powder diffraction only, and, because of the limitations of the powder method, there was little promise of unequivocal structure determinations of high pressure phases, except for those of the utmost simplicity. Some high pressure polymorphs can be stabilized by quenching to low temperatures while still under pressure and then studied by powder and single crystal x-ray diffraction techniques. This technique was employed whenever possible, but only a limited number of known high pressure polymorphs are quenchable in the metastable state. So, there was a critical need for producing and studying stable single crystals of high pressure polymorphs by x-ray diffraction, which meant that the crystal had to be studied in its *P,T* stability field.

In 1964, single crystal x-ray diffraction methods were still basically film techniques because computer driven automatic diffractometers were just being developed and not at all in common use. This was the situation in the Crystallography Section, for single crystal work was performed with Weissenberg and Buerger-type precession cameras. The precession camera was chosen for our investigation because, unlike the Weissenberg instrument, it had space available to accommodate the pressure cell and its design was simple to modify.

**Fig. 9:**
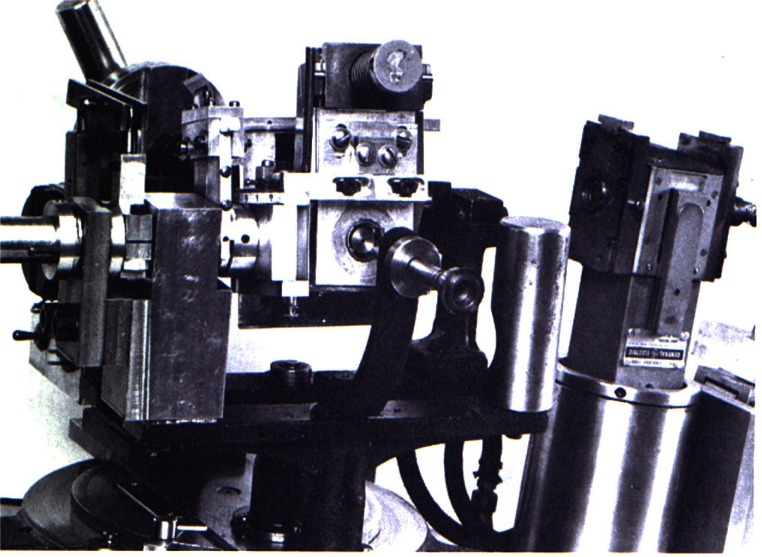
The original Buerger-type precession camera modified by C. E. Weir in 1964 for single crystal x-ray diffraction studies at high pressures. A specially designed diamond cell with very large windows was used to carry out these very first test experiments. As with the first diamond cell shown in Fig. 2, the modifications to this precession camera were crudely made for this exploratory work using simple machine tools available in the laboratory.

**Fig. 10:**
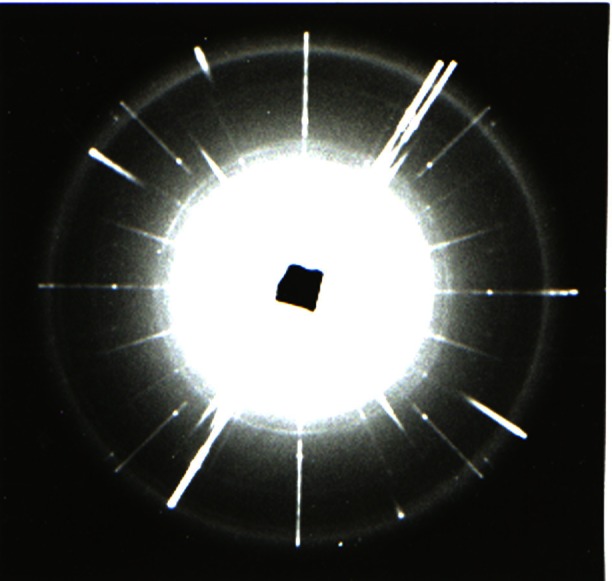
One of the very first precession patterns (Pd-filtered Ag radiation, *µ* = 16°) of the *hk0* level of ice VI at room temperature and 0.96 GPa (9.6 kbar) taken with the instrument shown in Fig. 9. The heavy diagonal streaks are from the single crystal diamonds and the rings are from the metal gasket. This x-ray precession pattern one of the very first ever taken of a single crystal at high pressure.

**Fig. 11:**
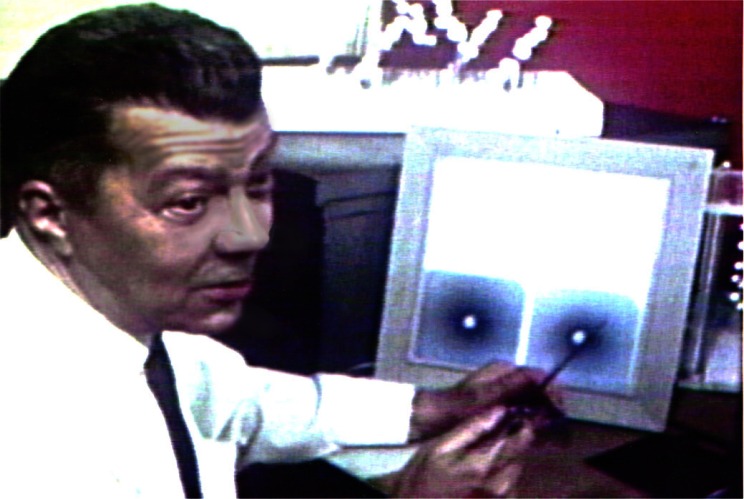
A photograph of C. E. Weir examining two precession films of ice VI, from “Crystal Structures at High Pressures,” a movie produced at NBS in 1970. The 40 minute color film describes the development of the single crystal DAC technique for high pressure studies.

To test our notion that single crystal studies might be possible at high pressures, we carried out initial experiments utilizing a typical lever-arm pressure cell with stainless steel pistons modified to include a 90° apex angle conical aperture, thus permitting a relatively large sampling of reciprocal space. At the same time, however, the cell’s pressure capability was reduced to less than 5 GPa because the large conical aperture provided less load-bearing support for the anvils. The pressure cell, mounted on a crudely modified Buerger-type precession camera, is shown in Fig. 9. In its early stages of development, Weir altered the precession camera himself using the same basic machine tools he used several years earlier to fabricate the original infrared pressure cell, thus, its rudimentary appearance. The first experiments were carried out on a single crystal of ice VI grown from high purity distilled water at about 0.9 GPa and RT. Palladium-filtered Ag radiation was used to minimize absorption effects (largely due to the diamond anvils) and also to decrease the size of the reciprocal lattice permitting more data to be obtained. These early experiments with the crudely modified precession camera demonstrated unequivocally that useful x-ray patterns could be obtained from the ice VI single crystal in a DAC [8,9]. Fig. 10 shows one of the very first precession patterns (*hk0* level) obtained for ice VI with this camera, and Fig. 11 is a photograph of C. E. Weir examining two precession films of ice VI taken at NBS in 1969. Precession patterns containing reciprocal lattice parameters, a* and b*, were observed, but none containing c* were obtained because of the preferential direction of growth of the crystal and the restricted movement of the pressure cell itself. The cell constants for ice VI at 0.9 GPa and RT, derived from 30 observed unique reflections (the first ever obtained from a crystal *in situ*) listed in Table 1, were *a* = (8.38±0.05) Å, *b* = (6.17±0.05) Å, *c* = (8.90±0.15) Å. Errors are smaller for *a* and *b* than for *c* because *c* was measured indirectly from cone-axis and mixed-index patterns. The observed systematic absences were *hk0, h* = 2*n* + 1 and *0k0, k* = 2*n* + 1 and indicated the orthorhombic aspect P**a. The cell constants found in this work successfully indexed previously reported, but unindexed, reflections obtained from metastable polycrystalline ice VI at 90 K and ambient pressure.

Although a complete space group and structure had not been determined, the pioneering work with ice VI, and later ice VII, demonstrated for the first time that useful x-ray intensity data could be obtained at high pressures from single crystals grown and maintained in a diamond cell at RT. However, the results also indicated a critical weakness of the method, i.e., all of reciprocal space could not be examined because of interference from opaque (to x rays) parts of the pressure cell. To observe all of reciprocal space, it was necessary to re-grow the crystal, perhaps several times, in order to obtain enough orientations to achieve this. Through this tedious procedure, it might be possible to examine all of reciprocal space to permit a complete structure analysis of an unknown high pressure polymorph. Despite this limitation the results appeared promising, so we proceeded to modify our design and to implement new procedures to improve the quality and quantity of data obtained by the method.

**Fig. 12:**
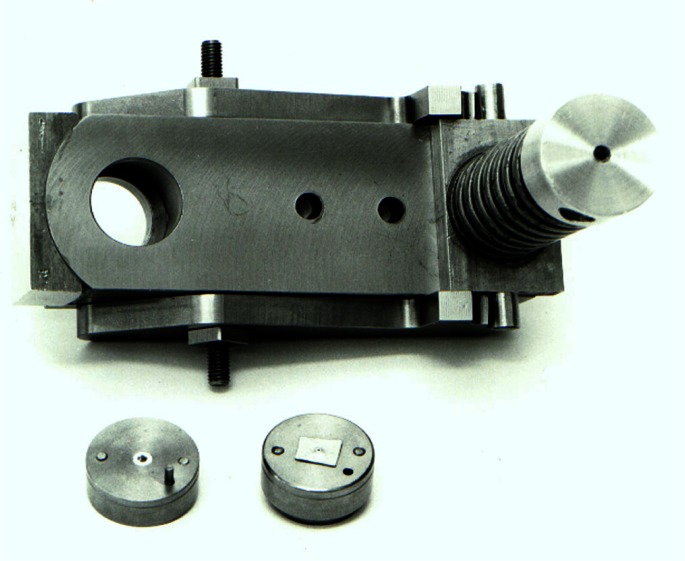
The original NBS Be diamond cell (disassembled) made in 1964 was designed to be used with the modified Buerger precession camera for high pressure single crystal x-ray diffraction studies.

**Fig. 13:**
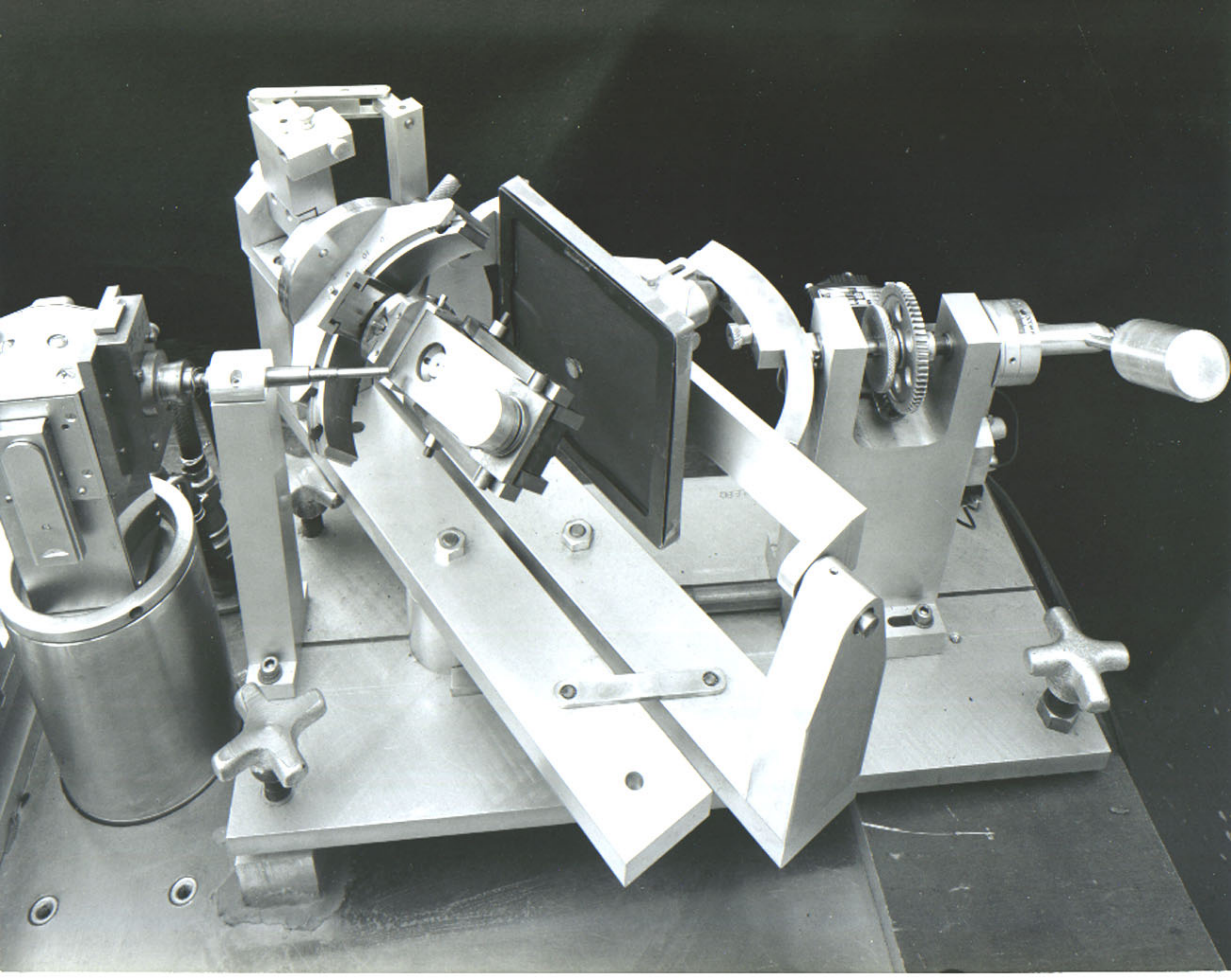
The Be diamond cell is mounted on a Buerger-type precession camera built especially for this cell. The overall dimensions of the camera were determined by (1) the dimensions of the Be cell, and (2) the large goniometer head, necessary to support the weight of the Be cell and also to provide the large angular corrections required for orienting the crystal in the cell. Because of the Be cell’s large size and special camera requirements, and, at that time, the absence of pressure measurement capability, the system was not used widely.

**Fig. 14:**
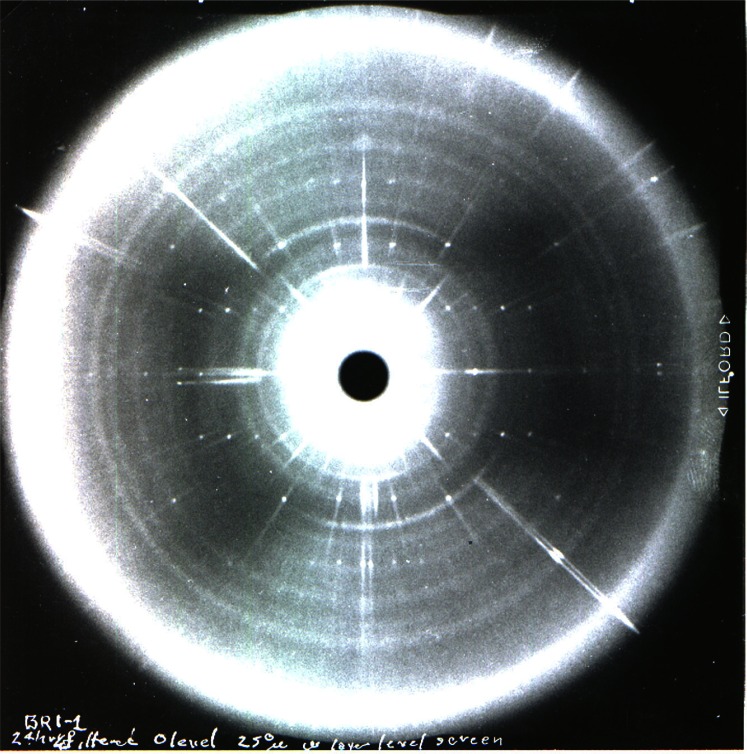
A precession pattern of a single crystal of bromine (0 level, *µ* = 25°) grown and maintained under a pressure of about 1 GPa(10 kbar). It was taken with the apparatus shown in Fig. 12 to provide intensity data on a known crystal for absorption correction analysis.

**Fig. 15:**
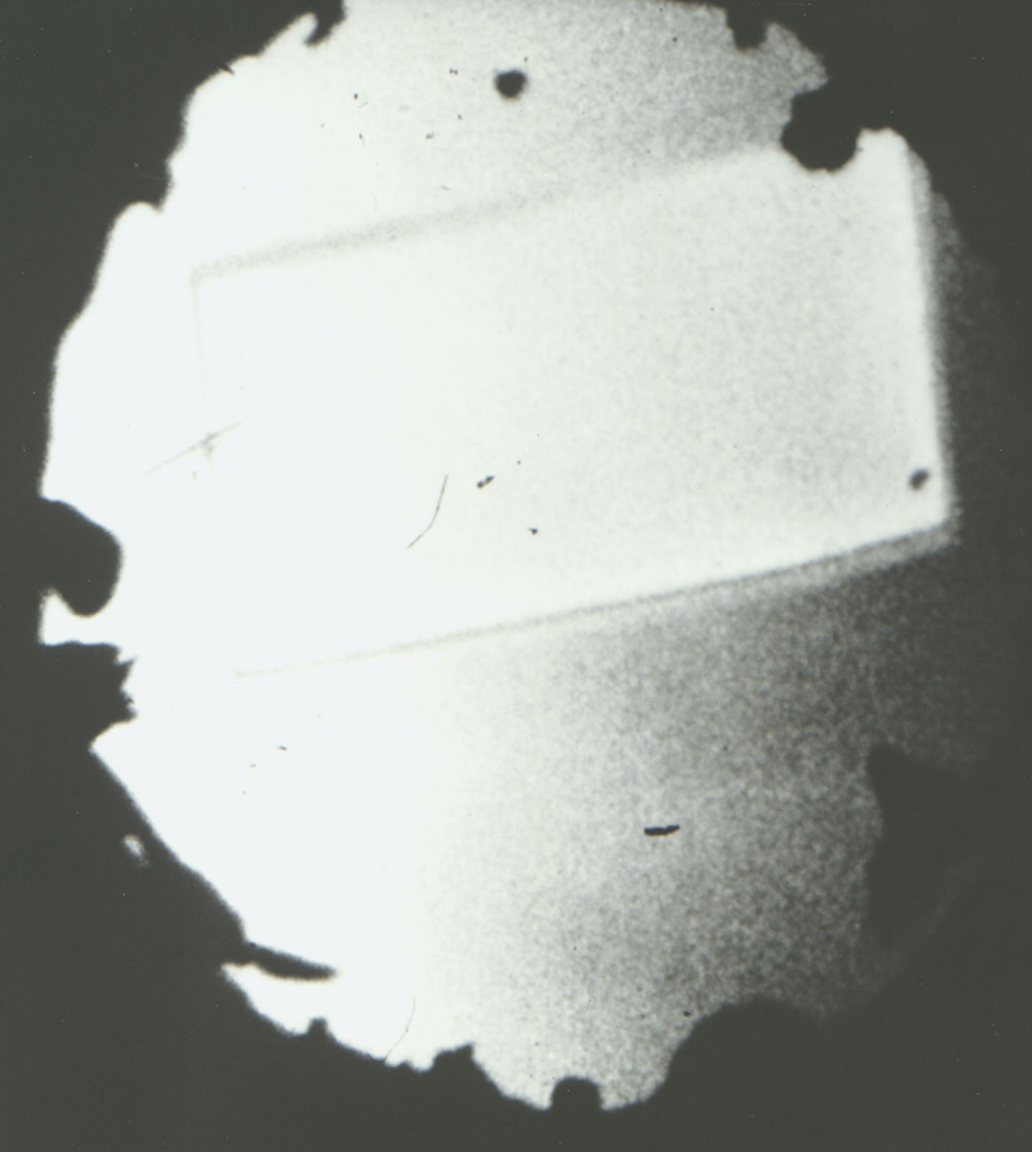
A single crystal of benzene II in equilibrium with liquid at about 310 °C and 3.0 GPa (30 kbar), showing well-defined crystal morphology as viewed through the window of the Be DAC. This crystal, retrieved to room temperature at 2.5 GPa (25 kbar), was used in the first crystal structure determination of an unknown, utilizing the apparatus shown in Fig. 12.

Our first improvement involved replacing the DAC with one fabricated almost entirely from Be metal (except for the load-generating spring and screw) to minimize absorption effects and to increase the range of orientation of the crystal sampled by the x-ray beam. The Be DAC, shown disassembled in Fig. 12, closely followed the original lever-arm design. Also, a new precession camera with a massive goniometer head was built to support the weight of the Be cell and to permit large translational and angular ranges for centering and orienting the crystal [10]. This new precession camera with the Be cell mounted on the very large goniometer head is shown in Fig. 13. To obtain more accurate intensity data, a procedure, originally developed by A. Santoro for evaluating the absorption corrections for complex cases, i.e., where other objects besides the crystal take part in the absorption, was applied to the Be cell [11]. The validity of the procedure was tested on intensity data obtained from a single crystal of bromine at 1.0 GPa and RT. Fig. 14 shows a precession pattern for bromine (0 level, *µ* = 25°) taken with this new equipment. The calculated absorption corrections were considered satisfactory and should be applied. The residual *R* index (a measure of the correctness of the structure) was 0.091 and the overall conclusion was that, in the case of simple structures such as bromine, useful structural information could be obtained. The first crystal structure determination of an unknown utilizing this method was a high pressure form of benzene (C_6_H_6_ II) at 2.5 GPa and RT [12]. Fig. 15 shows a single crystal of benzene II with well defined morphology in equilibrium with its liquid in a gasketed DAC at about 3 GPa and 310 °C, the *P,T* conditions necessary for growing the crystal. A total of seventy-two reflections were observed from thirteen zero-level and one upper-level photographs of the same crystal which could be indexed as a monoclinic unit cell with *a* = (5.417±0.005) Å, *b* = (5.376±0.019) Å, *c* = (7.532±0.007) Å, *β* = 110.00°±0.08°. After applying the absorption correction procedure for the Be cell, the 72 corrected intensities were reduced to a single relative scale by the use of common reflections. Equivalent reflections were averaged to give a set of 19 unique intensities for the same crystal. The observed extinctions are consistent with the space group *P*2_1_/*c* with two molecules per unit cell. Assuming *Z* = 2, the density of benzene II determined at RT and 2.5 GPa was 1.258 g cm^−3^. As demonstrated in Table 2, a comparison of the crystal data for benzene I and II, there is a significant increase in density of benzene II because of the pressure effect.

**Fig. 16:**
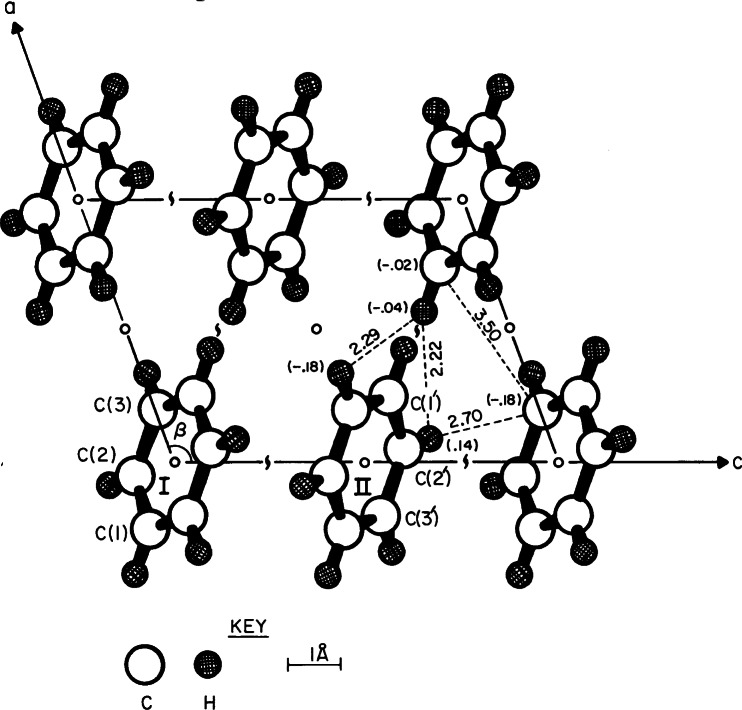
The projection of the structure of benzene II down the b-axis of the monoclinic cell (space group *P*2_1_/*c*). The intermolecular distances of closest approach are given for H⋯H, C⋯H, and C⋯C interactions. The *y* coordinate for the respective atoms is indicated in parentheses.

Because of the limited number of unique reflections (only 19), conventional strategies to solve the structure were not possible. Consequently, a different approach was devised by A. Mighell to solve the structure. The procedure involved developing a computer program to generate all physically possible structures (excluding those with unreasonable closest-approach distances) by rotating the benzene ring located on a special position in the unit cell. Three orthogonal rotation axes, *α*, *β*, and *γ*, (*α* and *β* coincident with *a* and *b* unit cell parameters, respectively, and *γ*, 20° from the c parameter in the *XZ* plane) were used to generate ring orientations and the structure was built-up on the basis of the space group symmetry requirements. To minimize computer run-time, a unique angular range much less than 0–2π was determined for the rotations in the order *γ*, *α*, and *β*. The ring was rotated throughout the unique range in 4° increments. To further minimize computer run-time, exclusion parameters were set to eliminate structures with unacceptable intermolecular distances of closest-approach. For each orientation, a scale factor and 2 reliability factors (*R*) were calculated. One (*R*_1_) was the conventional value, and the other (*R*_2_), a value which equally weights the reflections because the limited amount of data may give a low *R*_1_ value with some very poor individual agreements, especially for weaker reflections. Both *R* values gave minima with the same angular coodinates, but *R*_2_ was more sensitive and gave a sharper minimum. The final structure was determined with a 1° angular scan of *α*, *β*, and *γ*. Observed and calculated structure factors, listed in Table 3, show reasonably good agreement along with additional reflections for which the intensity was less than the background. The b-axis projection of the monoclinic structure (P2_1_/*c*) with some intermolecular distances of closest approach is shown in Fig. 16.

Following the structure work on benzene II, the technique was used at NBS to obtain unit cell and space group data on high-pressure forms of C_6_H_6_, CS_2_, Br_2_, CCl_4_, and KNO_3_ [13]. The results of those studies were reported for the first time and are listed in Table 4. We made several useful observations during those experiments. In most instances, for liquid-solid transitions, crystals grew in a fixed orientation with respect to the cell. When the crystal was small, it could and often did move around in its liquid, but, as it grew larger, it reoriented and the final orientation of the large crystal was invariably the same. It appeared that growth in a prescribed crystallographic direction predominated and effectively controlled the orientation in the confining volume. For crystals grown from solid-solid transitions, however, this behavior did not apply, as in the case of KNO_3_. In all of these cases, however, the pressure was not of primary importance and was considered as little more than an environmental parameter required to produce the phase of interest.

**Fig. 17:**
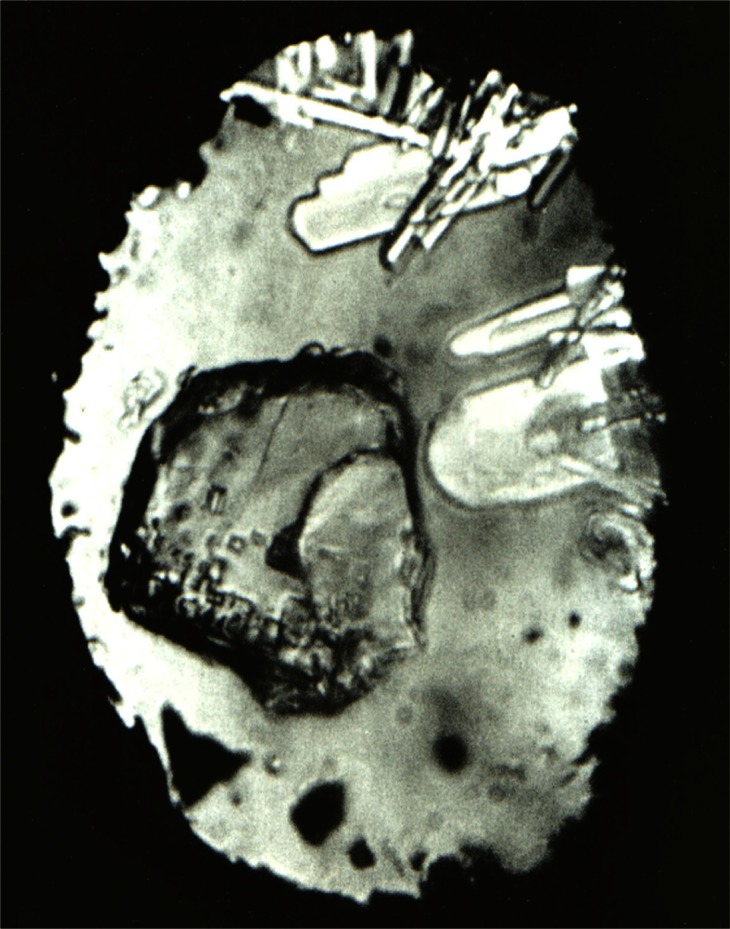
A single crystal of lead azide at approximately 2.2 GPa (22 kbar) and room temperature as viewed through the diamond window of the Be pressure cell. The matrix surrounding the crystal is liquid ethanol in equilibrium with multiple crystals of ethanol to the right and top edges of the picture.

The situation, however, was quite different in later single crystal work involving the measurement of anisotropic and volume compressibilities. For that work, a knowledge of pressure was essential. Six inorganic azides were studied: *α* lead azide, *β* lead azide, barium azide, potassium azide, sodium azide and thallium azide, all highly energetic materials [14]. Compressibility measurements on such unstable materials had never been done before and were not seriously considered because of the danger of explosion until the development of this single crystal high pressure x-ray technique. The small crystal required in this method made this danger almost negligible. Prior to the advent of the DAC, measurements to obtain compressibility data could only be made with relatively large sample-volume presses and such measurements are rare because the potential for violent detonation is too great. Thus, these measurements on the azide compounds at NBS utilizing the DAC made a great impact in the study of energetic materials at high pressures. Although the equipment was intended primarily for structure studies, it seemed to us that it could be used also to measure lattice parameters as a function of pressure, provided the pressure could be defined adequately. The pressures were estimated using a fixed-point scale based on the known freezing points of n-hexane and ethanol, 1.04 GPa and 2.22 GPa, respectively. The desired crystal was cemented in place on one of the diamond anvils and the gasket was filled with a hydrostatic liquid and sealed by the application of pressure. The pressure was increased slowly until the liquid partially crystallized as shown in Fig. 17 for a single crystal of Pb(N_3_)_2_ at 2.2 GPa and RT. The equilibrium between liquid and solid served to specify the pressure from the reported *P-T* behavior of the liquidus curve. The loaded cell was mounted on the precession camera at RT and as many reciprocal lattice planes as possible were recorded on film. A phase transition in thallium azide was discovered at a pressure between the freezing pressure of chloroform (0.54 GPa) and n-decane (0.30 GPa). Table 5 lists the results of those measurements which were being reported for the first time on the azide compounds.

**Fig. 18:**
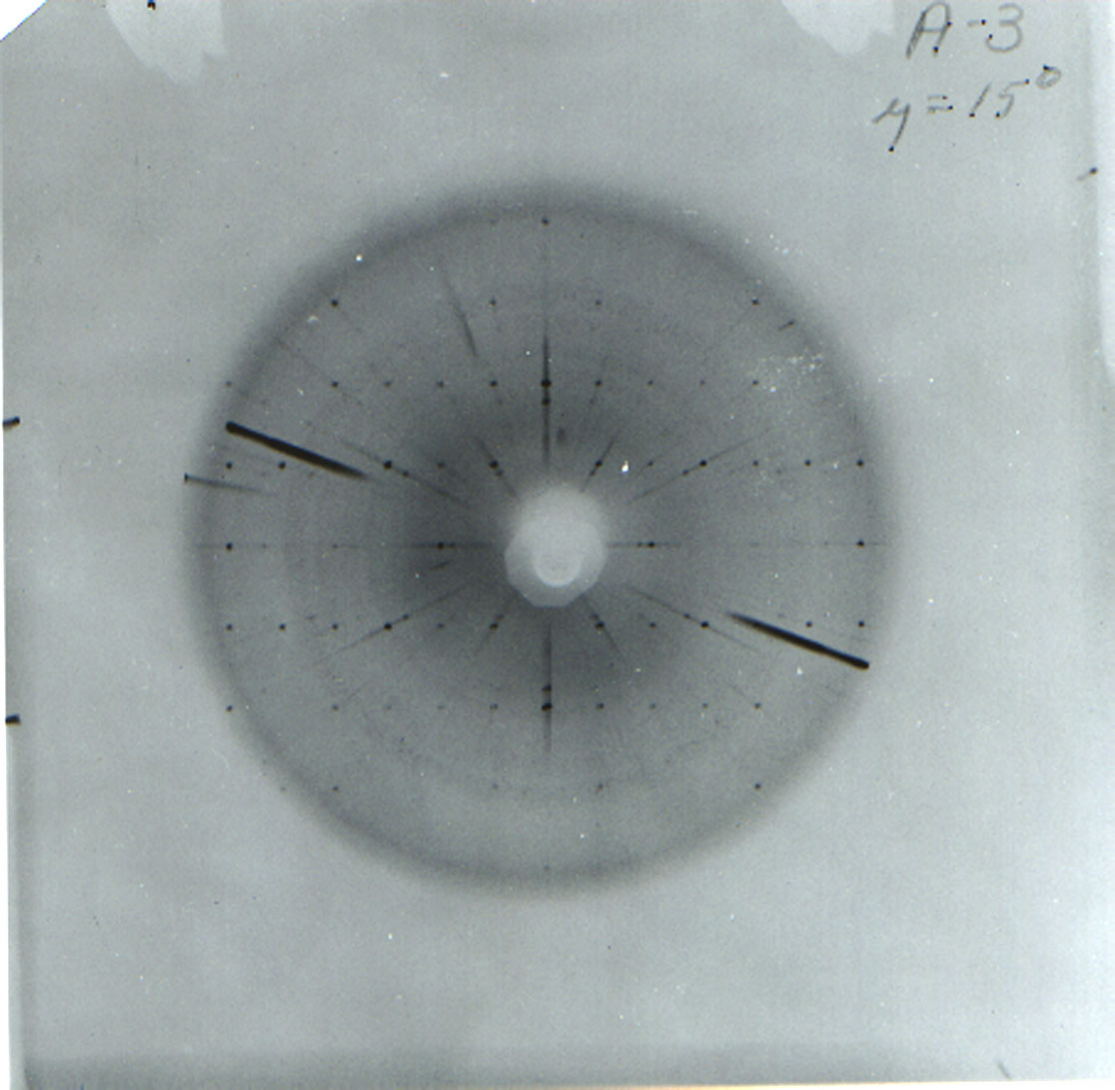
A typical zero-level precession pattern (Mo K radiation, Zr-filtered, *µ* = 15°, 23 h exposure) obtained for carbon tetrachloride III at approximately 1 GPa (10 kbar) and room temperature. The very small well-defined spots forming a regular array (c* is horizontal and b* is vertical in the monoclinic unit cell) are due to diffraction from the single crystal of CCl_4_ III. Diffraction from the two diamond crystals appear as large dark radial streaks which may interfere with the desired diffraction from the CCl_4_ crystal.

**Fig. 19:**
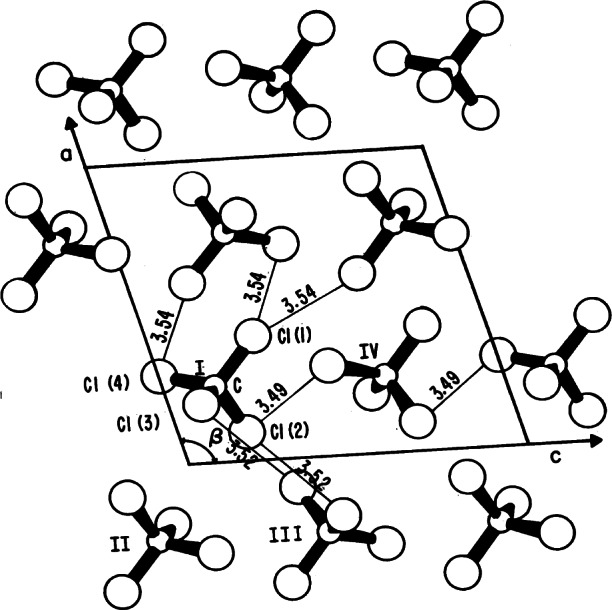
The *b* axis projection of the monoclinic cell of the CCl_4_ III structure (space group *P*2_1_/*c*). Molecule I is related to II, III, and IV by a center of symmetry, a twofold screw axis and a *c* glide. Minimum closest approach of nonbonded chlorine atoms and other short distances are in Å. The determination of the structure of CCl_4_ III was the second unknown crystal determined by this precession method.

The second unknown structure determined by this method was a high pressure form of carbon tetrachloride (CCl_4_ III) at 1.0 GPa and RT [15]. A typical zero level pattern (Zr-filtered Mo radiation, *µ* = 15°, 23 h exposure) taken of CCl_4_ III at 1 GPa and RT is shown in Fig. 18. Carbon tetrachloride III crystallizes in the monoclinic system (*P*2_1_/*c*, *Z* = 4) with unit cell dimensions *a*=(9.079±0.012)Å, *b*=(5.764±0.003)Å, *c* = (9.201±0.004) Å, and *β*= 104.29°±0.05°. Using four molecules per unit cell, the calculated density was 2.19 g cm^−3^ and agreed well with reported values for those conditions of pressure and temperature. The *R* value (0.0951) for CCl_4_ III (SnBr_4_-type structure) was approximately the same as that found for benzene II. Table 6 lists the 52 unique observed and calculated structure factors for the phase. The *b*-axis projection of the monoclinic cell of the CCl_4_ III structure is shown in Fig. 19. It is interesting to note that the *R*-value obtained for the CCl4 III structure based on 52 unique reflections was no better than that obtained for benzene II which used a set of 19 unique intensities. It appeared that *R* values in the 9 % range were all that one could expect from this film technique. The crystal structure of CCl_4_ III is isostructural with the structures reported for SnBr_4_, TiCl_4_, TiBr_4_, and probably SnCl_4_. Table 7 lists the lattice constants of these five tetrahalogens exhibiting the SnBr_4_-type structure. Similarities exist in the unit cell dimensions as well as their ratios with the differences reflecting the relative size difference in the atoms of the various substances.

In the CCl_4_ III structure, chlorine atoms occupy a distorted hexagonal-close-packed arrangement with the distortion arising from the marked difference between inter- and intra-molecular nonbonded chlorine-chlorine distances which are 3.49 Å and 2.88 Å, respectively. Because this structure deviates significantly from a close-packed arrangement, it was thought that a more compact phase of CCl_4_ must exist and should be found at higher pressures. Indeed, its existence was confirmed by optical polarizing microscopy observations with the DAC at pressures above 1.5 GPa at RT, and the new phase was named CCl_4_ IV. The crystal under polarized light exhibited no detectable birefringence suggesting that it belonged to the cubic system. Further work with the single crystal high pressure x-ray precession camera identified the space group (*Pa* 3) with *a* = (9.334±0.008) Å, determined from 27 reflections. The data were obtained at 2.4 GPa and RT. The calculated density, assuming eight molecules per unit cell, was 2.513 g cm^−3^, and, as expected, was greater than the value for CCl_4_ III (2.190 g cm^−3^) measured at 1.0 GPa. By analogy, we assumed the structure to be an isotype of the SnI_4_ structure. No further work was done on CCl_4_ IV because more pressing priorities arose related to the development of a spectroscopic method to measure sample pressure in the DAC.

The high pressure x-ray precession film method was not used widely because of the Be cell’s large size and expensive fabrication costs. Also, the special camera requirements and large goniometer head demanded the construction of an expensive precession instrument, a task which many scientists declined to undertake. Another contributing factor to its lack of acceptance was the absence of a reliable, convenient pressure measurement capability for the sample in a DAC. However, the method demonstrated its usefulness and served as the foundation for later more advanced work in the early-to-mid 1970s in other laboratories. The basic opposed-anvil cell design underwent miniaturization and further simplification and was adapted to automatic diffractometers for single crystal x-ray diffraction studies, rapidly displacing the earlier precession film method. Another contributing factor to the acceleration of high pressure single crystal x-ray diffraction studies was the introduction of the ruby fluorescence technique for pressure measurement by NBS in 1971.

## 4. The Ruby Fluorescence Method of Pressure Measurement

By 1971, the DAC had undergone several stages of refinement and had been adopted by other laboratories in the U.S. and abroad. Still, it was not fully appreciated by many scientists because there remained a serious deficiency with the instrument. There was no convenient, rapid and reliable method for measuring the sample pressure. This situation is reminiscent of the statement made by the famous 19th century physicist, William Thomson (better known to us as Lord Kelvin). In one of his many lectures, he said: “When you can measure what you are speaking about, and express it in numbers, you know something about it; but when you cannot measure it, when you cannot express it in numbers, your knowledge is of a meager and unsatisfactory kind: it may be the beginning of knowledge, but you have scarcely, in your thoughts, advanced to the stage of science” [16].

Such was the case with the DAC before 1971. Pressures were either calculated (force per unit area) or measured from compression data (utilizing an equation of state, e.g., sodium chloride) obtained by an x-ray powder diffraction film method. The former procedure was extremely inaccurate because it was difficult to ascertain the distribution of the applied load over the gasket/sample area. The latter procedure, although reliable, was tedious and time consuming, often requiring as many as 15 h to make one pressure measurement. In that time frame, many factors could influence and change the assumed conditions under which the desired measurement was made. Consequently, acceptance of the DAC as a tool in high pressure research was somewhat limited in 1970. It was used primarily in laboratories engaged in research of geological interest where very high pressures simulating the earth’s interior were desired, while the accuracy in the value of pressure was not yet considered of primary importance.

**Fig. 20:**
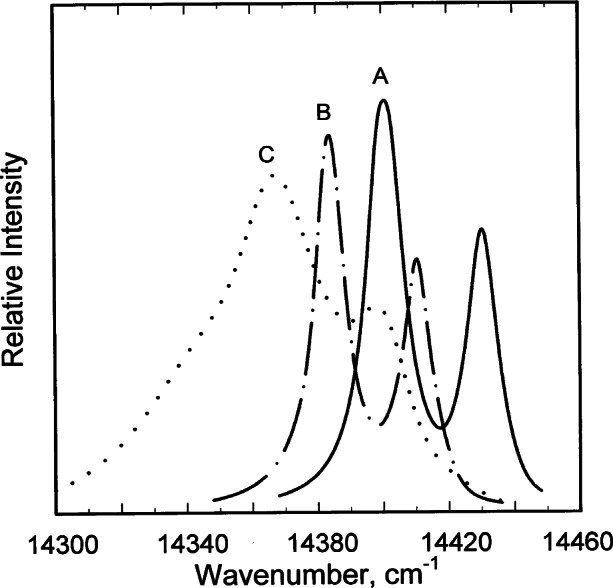
The R-line luminescence spectra of a crystal of ruby in the diamond anvil cell at room temperature. Ruby crystal: at ambient pressure (A); at about 2.23 GPa (22.3 kbar) hydrostatic pressure (B); and at an average nonhydrostatic pressure of about 4 GPa (40 kbar) (C). Effects of a hydrostatic pressure-shift and line-sharpening (A)-to- (B), and nonhydrostatic pressure-shift and line-broadening (A)-to- (C), are illustrated in this figure. The pressure-shift is to lower energy (toward the red) with increasing pressure.

This situation changed dramatically when the high pressure group in the CS, under constant encouragement to develop a rapid, convenient and reliable method for measuring pressures in the DAC, achieved a breakthrough in 1971. The problem was discussed one day in the NBS cafeteria among John B. Wachtman, Jr. (Chief of the Inorganic Materials Division), Stanley Block (Chief of the Crystallography Section), and J. Dean Barnett, a guest scientist at NBS on sabbatical leave from Brigham Young University. While having lunch together, they discussed possible techniques for measuring pressure in the DAC. Wachtman suggested various methods, all of which had been considered already and found to be unsuitable. Finally, Wachtman asked, “Have you considered fluorescent spectroscopy” As it happened, they had not, but replied they would look into it. Encouraged by this meeting, Stanley Block, Dean Barnett, Gasper Piermarini, and Richard Forman began measuring the pressure-dependence of several fluorescing materials that happened to be on the shelves in Forman’s spectroscopy laboratory and in H. Parker’s and W. Brauer’s crystal growing laboratory. Those materials included ruby (Al_2_O_3_), YAlO_3_, YAG, MgO, and a few others. While several materials were found to have a readily measurable pressure dependence, ruby exhibited by far the most promising results. Ruby’s main fluorescence lines (the R_1_R_2_ doublet) were intense and sharp, and the lines shifted measurably toward the red with increasing pressure [17]. Fig. 20 shows the effect of 2.2 GPa (hydrostatic) and 4 GPa (nonhydrostatic) compared with ambient *P, T* conditions. Significantly, it was observed that pressure could be detected *in situ* using only a very small amount of ruby crystal as the internal pressure sensor, comprising only 1 % of the available volume under pressure. Further, because ruby is generally chemically inert, it can be present in the sample chamber as an internal pressure sensor without interfering with the desired measurement on the specimen.

### 4.1 Pressure Calibration of the Ruby R_1_ Line-Shift

**Fig. 21:**
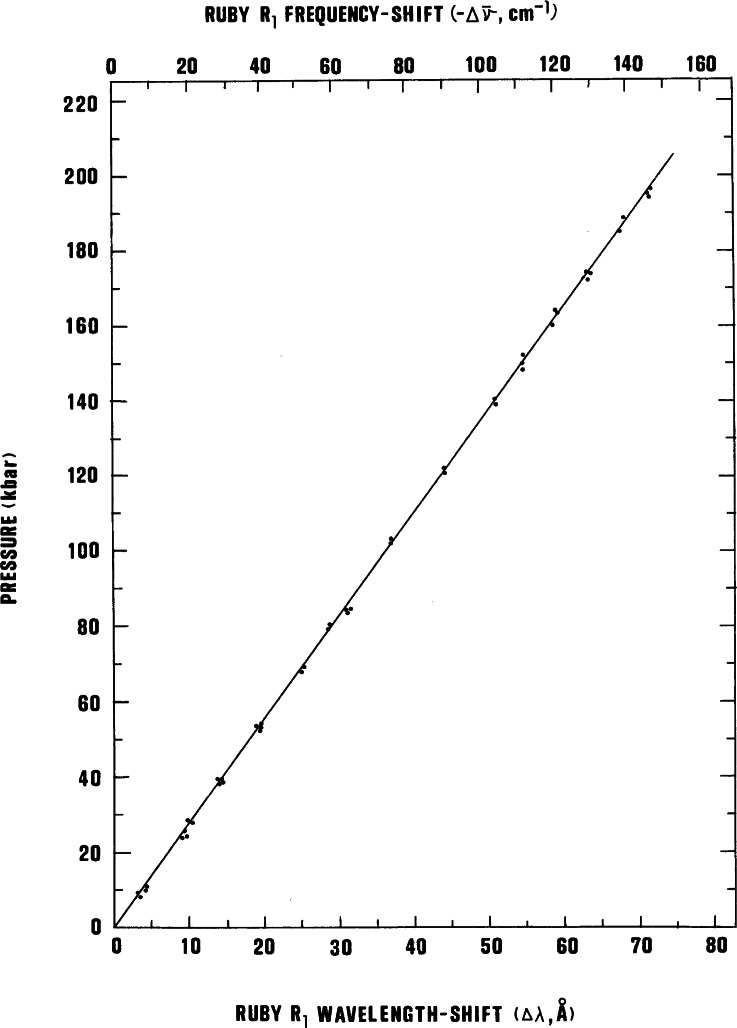
A graph of the pressure dependence at 25 °C of the ruby R_1_ fluorescence line at 6942 Å as a function of wavelength (bottom) and frequency (top). Pressures are based on the Decker equation of state for NaCl.

**Fig. 22:**
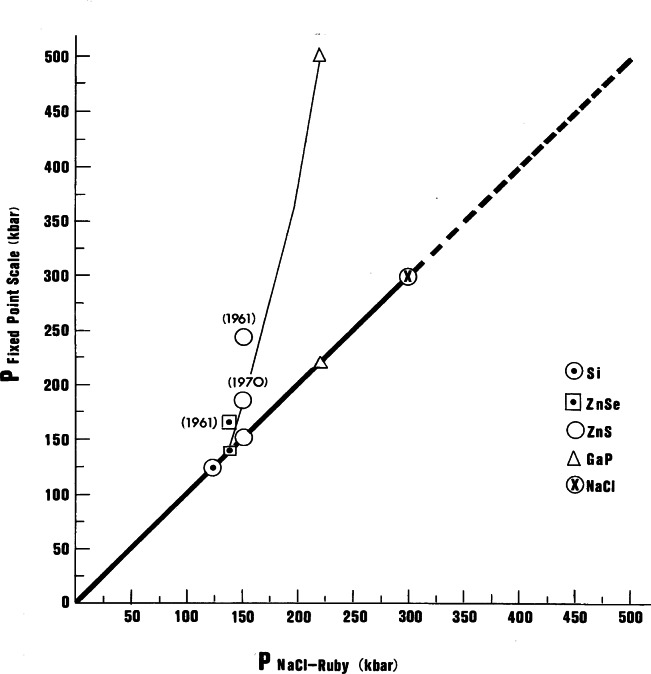
This graph compares the transition pressures (for the materials indicated) based on the fixed-point scale (thin line) and ruby-NaCl scale (heavy line). The two scales begin to diverge above approximately 14.5 GPa (145 kbar), with the fixed-point scale defining the higher pressures. The points identified with “1961” refer to the original fixed-point scale and those with “1970” refer to the 1970 revised scale. The phase transitions associated with the fixed-point scale are based mainly on electrical resistance measurements on samples subjected to pressure in large scale presses. In such an arrangement, there is great uncertainty in estimating pressures because they are based on poorly characterized complex force/area calculations. Thus, in the higher pressure regime where sample configuration becomes greatly distorted from the ideal, pressures were greatly overestimated. The ruby scale, on the other hand, is based on an internal pressure sensor (a chip of ruby crystal in contact with the sample) and pressures are derived from the Decker equation of state for NaCl.

Recognizing the significance of these observations, the group concluded it was of paramount importance to calibrate the R-line shift against reliable pressure values. The calibration was accomplished using an x-ray powder diffraction film method to measure the compression of NaCl for a measured shift in the wavelength of the ruby R_1_-line [18]. The corresponding pressure was calculated from the compression data utilizing an established equation of state for NaCl. The R_1_-line shift was found to be linear with pressure, (0.2740±0.0016 GPa Å^−1^), up to about 19.5 GPa, shown graphically in Fig. 21. In subsequent work, the linearity was confirmed to about 29 GPa [19]. In 1970, a fixed-point pressure scale, based on a series of well-characterized phase transitions, was widely used and accepted for estimating pressures in most high pressure experiments performed at that time. A test of the validity of the fixed-point scale was made by measuring the transition pressures of the fixed-points using the linear scale of the ruby technique and comparing these measured pressures with the accepted values. The results showed that the fixed-point scale required serious downward revision by a factor of two at 50 GPa. The divergence of the two scales is shown graphically in Fig. 22. The success of this calibration quickly established the ruby scale as a secondary pressure standard, and, it soon became the *de facto* standard for measuring pressures in a DAC in all kinds of experiments including both powder and single crystal x-ray diffraction.

**Fig. 23:**
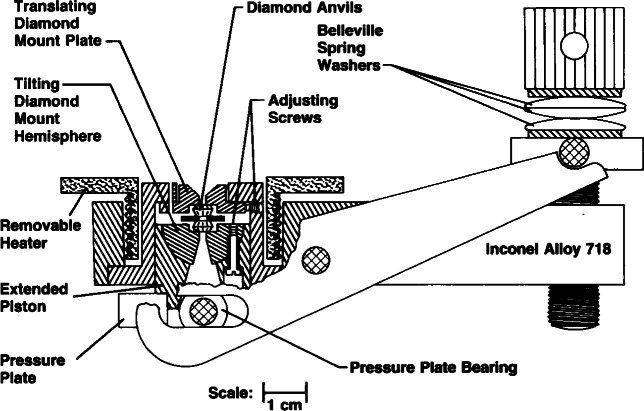
Cutaway cross section drawing of an improved diamond cell for use at high temperatures. This cell was designed and built at NBS in 1972, and was the first DAC to be used to study pressure effects on materials at elevated temperatures up to 600 °C. The cell was fabricated from a high temperature nickel-base alloy and features a removable coil-type heater surrounding the anvil assembly for producing static high temperatures.

Shortly after its calibration, the pressure-dependence of the ruby-shift was found to be essentially independent of temperature within the limits of uncertainty of the measurement at that time, so it was possible to use it to measure pressure at temperatures other than RT [20]. It was particularly useful at elevated temperatures where many high pressure phenomena were observed. Consequently, a specially designed DAC for use at elevated temperatures was made at NBS in 1972. A cross section of the design of that instrument is shown in Fig. 23. It was first used at NBS to study many materials to temperatures as high as 600 °C, but the quality of the pressure measurement rapidly deteriorated above 300 °C.

### 4.2 Evaluating Stresses in Solid and Liquid Pressure-Transmitting Media

In the years following the introduction of the ruby technique, the group at NBS also developed techniques to obtain useful information from the pressure-dependence of the ruby R-lines concerning the stress conditions in the gasketed and ungasketed sample configurations, both widely used in a DAC at that time. Two methods to measure the nature of the stressed environments in compressed liquids and solids in a DAC were developed which provided extremely useful information on pressure-transmitting media used in x-ray diffraction experiments at high pressure, particularly for single crystal work. Method I uses finely powdered ruby crystals dispersed in a powdered sample. It was used to study stress distributions in both the gasketed or ungasketed configurations. The pressure is measured at several localized areas over the extent of the sample. This spatially sensitive technique, although time consuming, yields quantitative measurements of pressure gradients at different locations in the sample. Method II, generally used to study stress distributions in liquids, employs several small ruby crystals or just one large crystal fragment in the gasketed sample configuration. The spectral line-width of the R_1_ peak emitted from an extended region of the sample chamber is measured. Under hydrostatic conditions, the line-width decreases slightly with increasing pressure, but increases dramatically when the ruby senses nonhydrostaticity in the medium. Method II yields only a qualitative measure of the pressure gradient, but is much more rapid that Method I.

**Fig. 24:**
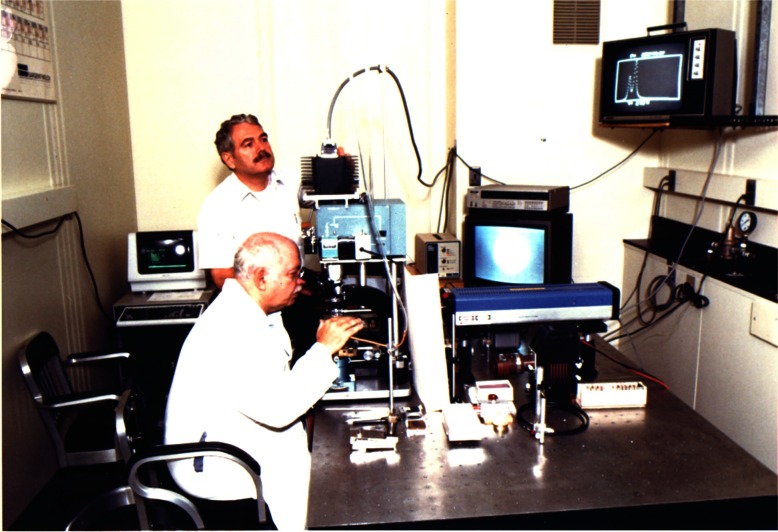
A photograph of the pressure measuring system with a DAC mounted on the microscope stage. In the initial stages of its development, a photomultiplier tube and strip chart recorder were used to measure the fluorescence intensity. Also, a video camera was incorporated in the system to record pressure-induced phenomena on materials. In the improved version of the instrument shown here, with Block and Piermarini, a linear diode array, has replaced the photomultiplier tube and the pressure (wavelength) is determined utilizing computer software. The R-line intensity is partially displayed on the monitor in the upper right of the photograph.

**Fig. 25:**
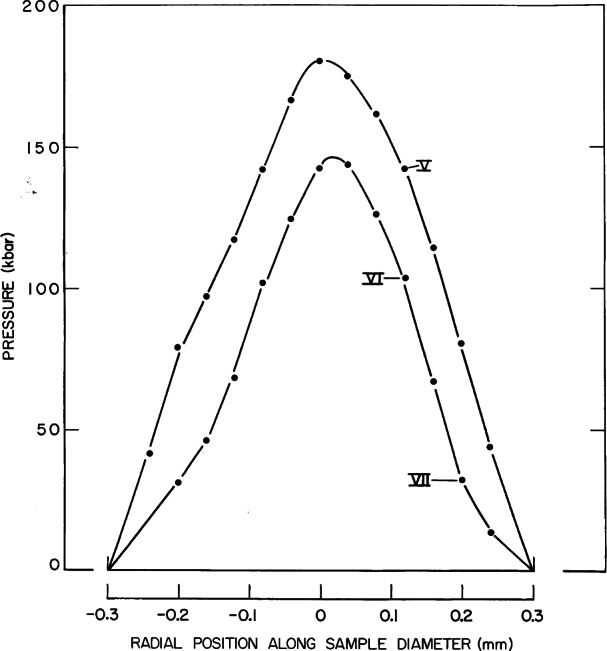
Pressure distributions in an ungasketed sample of powdered NaCl squeezed between the diamond anvils at two different applied loads.

Method I was used to study the stress distribution in an ungasketed powdered sample. The ungasketed sample is the simplest configuration, and was the first used with the DAC. It was found to be least desirable for achieving homogeneous stress. In this configuration, a powder dispersed with numerous minute crystals (1 µ to 10 µ) of ruby is simply squeezed between the two anvil flats, thinning down the powder until frictional forces prevent further flow. The distribution of stress in the compressed layer of powder can be determined from shift and broadening properties of the ruby R-lines. To illustrate, R-line spectra were measured from individual ruby particles in a NaCl/ruby powder pressed between the anvil flats using a microscope coupled to a specially constructed fluorescence wavelength measuring instrument. The instrument, the first of its kind, was designed and constructed by Barnett, Piermarini, and Block specifically to measure the wavelength-shift of the ruby R-lines [20]. It contained a special Eschelle grating which could measure pressure (wavelength) with a precision of ± 0.025 GPa under ideal conditions. A photograph with Block and Piermarini operating the instrument is shown in Fig. 24. Results, shown in Fig. 25, depict a parabolic distribution of stress along an anvil diameter for two different applied loads [21]. As expected, the results demonstrated clearly that in x-ray powder diffraction experiments where the sample is ungasketed, a large stress distribution is experienced by the sample, producing significant broadening in the diffraction rings. To minimize this undesired effect, a very small diameter (0.2 mm) collimated x-ray beam is used, allowing only the very center of the sample (top portion of the parabola where stresses are maximized and over a narrow range) to contribute to diffraction. With conventional x-ray sources available at that time, such finely collimated beams required long exposure times to get measurable diffraction patterns on film. Later, this situation changed dramatically with the advent of synchrotron radiation sources.

**Fig. 26:**
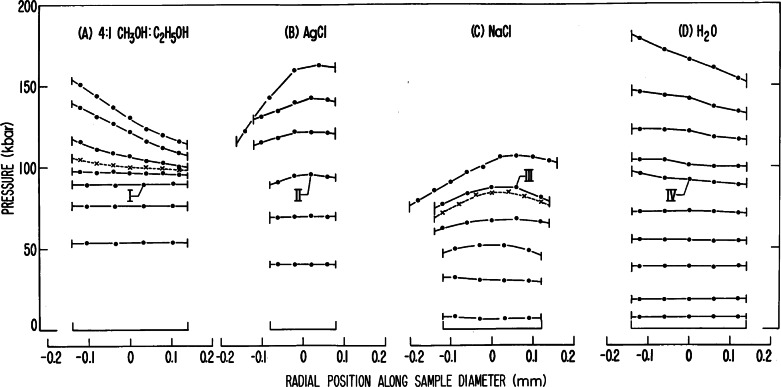
Pressure distributions in several materials enclosed in a gasketed diamond cell.

Methods I and II were used to determine the nature of the stress environment in powders, liquids, and combinations of both compressed in a gasketed DAC. Fig. 26 shows results obtained with Method I (line-shift) for increasingly applied loads on some important pressure-transmitting media materials: water, powdered NaCl, powdered AgCl, and a 4:1 (by volume) mixture of methanol:ethanol. Two, NaCl and AgCl, were studied because they were widely used at that time as a pressure-transmitting medium in solid-media high pressure systems (presses) because they were thought to produce a desired quasi hydrostatic environment. Water was studied because of general geological interest in this fundamental material. The methanol:ethanol mixture was studied because of its potential importance as a truly hydrostatic medium, exceeding by a large amount the limit for that time, (6.5 to 7.0) GPa, provided by 1:1 (by volume) pentane:isopentane. The results (Fig. 26) indicate that powdered NaCl is the least desirable of the four materials studied because it shows deviations in pressure at very low values, with differences becoming quite prominent above 4.0 GPa, while AgCl appears to be much better in achieving quasi hydrostatic conditions to much higher pressures. No gradient in pressure is indicated below 6.5 GPa. The results for H_2_O indicate an unexpected low gradient in pressure at 10 GPa. Water appears to be at least as good as and perhaps better than AgCl for providing a quasi hydrostatic environment in the 10 GPa range. No measurable pressure gradient was observed in 4:1 methanol:ethanol mixture until the pressure exceeded approximately 9.5 GPa, extending the hydrostatic limit significantly beyond the then current limit of (6.5 to 7.0) GPa. The work clearly demonstrated, as expected, that the gasketed configuration is far superior to the ungasketed sample for producing a hydrostatic or quasi hydrostatic environment in a DAC.

**Fig. 27:**
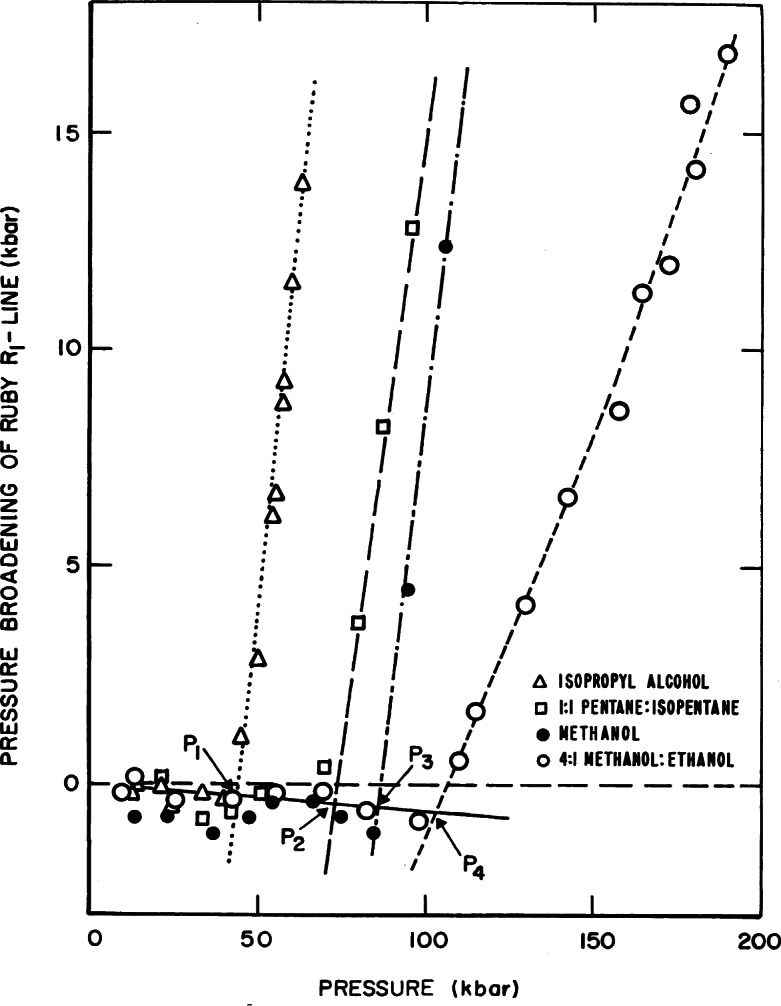
Effect of pressure on peak-width of the sharp ruby R_1_ fluorescence line for various pressure-transmitting liquids relative to the linewidth at ambient pressure.

**Fig. 28:**
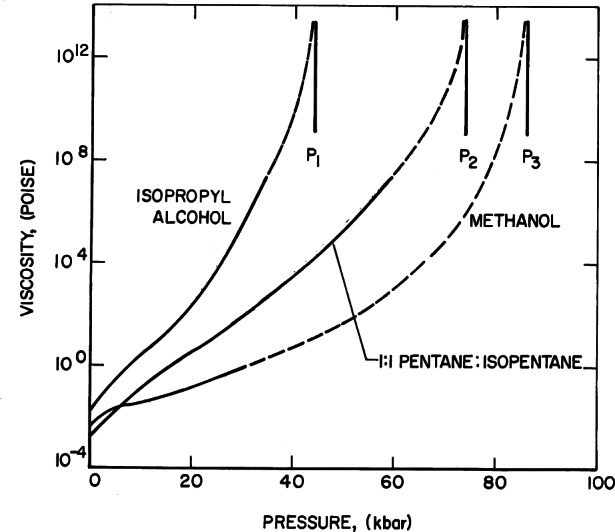
Extrapolations of reported viscosity data to measured glass transition pressures for isopropyl alcohol (P_1_), 1:1 pentane:isopentane (P_2_), and methanol (P_3_).

Method II (line broadening) was used to study the stress properties in many liquids. Fig. 27 shows line-broadening results for four liquids that do not crystallize easily at high pressures: isopropyl alcohol, 1:1 (by volume) pentane:isopentane, methanol, and 4:1 (by volume) methanol:ethanol. The data are plotted relative to the ambient pressure line-width. The general features of the curves are similar for each liquid. There is a slight decrease in line-width with increasing pressure followed by a sharp discontinuity which characterizes the pressures (*P*_1_, *P*_2_, *P*_3_, and *P*_4_) at which the ruby senses inhomogeneous stress. These discontinuities have been related to the glass-transition pressure for the given material. Because the liquid does not crystallize, its viscosity increases with pressure until it approaches 10^13^ P, the value generally accepted as the glass transition. These concepts were correlated by measurements of the pressure-dependence of viscosity of 4:1 methanol:ethanol and other liquids utilizing the DAC and a simple falling-ball Stokes technique as shown graphically in Fig. 28 [22, 23].

From a practical standpoint, the significance of the work on liquids established the fact that hydrostatic conditions could be achieved to pressures near 10 GPa, far in excess of the previously attainable limit of 7.0 GPa with 1:1 pentane:isopentane. However, it needs to be emphasized that even in liquids, time-dependent shear stresses do, indeed, exist and the rate of change in pressure must be carefully considered when the viscosity of these hydrostatic fluids exceeds roughly 10^10^ P. If changes in pressure are too large, then under these circumstances it is likely that plastic deformation will occur in low-shear-strength solid specimens as a result of time-dependent low magnitude stresses which are insensitive to the ruby monitor. This was an important result for x-ray diffraction experiments, particularly for single crystal work where the crystal must remain intact and without defects. While quality x-ray data could be obtained over a much larger pressure range with these fluids than previously, it was also important to be aware of the potential consequences of approaching the hydrostatic limit for a given medium too rapidly. Because the 4:1 methanol:ethanol mixture was easy to prepare and load into a gasketed DAC both for powder and single crystal x-ray diffraction work, it was widely used as a pressure-transmitting medium in early x-ray diffraction experiments, especially for single crystal x-ray diffraction where hydrostatic environments are essential for preventing shear in the crystal. In fact, for the reasons cited above, it is still often used today. Line broadening studies also led to the discovery of another pressure-transmitting liquid medium, 16:3:1 (by volume) methanol:ethanol:water, which extended the hydrostatic limit to 14.4 GPa [24]. It, too, was easy to use and consequently enjoyed wide popularity. It is still used today.

## 5. Other X-Ray Diffraction Techniques Used With the DAC

In the years following the development of the ruby technique, the high pressure group in the Crystallography Section developed two additional techniques for x-ray diffraction studies with the DAC: (1) an energy dispersive x-ray technique and (2) a single crystal Bond method. The energy dispersive technique was used in numerous applications including compressibility measurements, detection of pressure-induced phase transformations, identification of high pressure phases, and even some innovative applications. A few noteworthy examples follow. It was shown possible, for the first time, to determine atomic radial distribution functions (RDF) of amorphous materials contained in a DAC. The pressure dependencies to 10.5 GPa of RDFs for two amorphous materials, Fe-W and Ni-P were reported and critically evaluated [25]. However, it was noted that the limitations on the data imposed by the operating conditions were significant and correspondingly limited the results derived from that data. It was estimated that the distance of the first nearest-neighbor shell of atoms can be determined with an absolute accuracy of not better than 3 %. Energy dispersion was also used for the first time to study the effect of pressure on the crystal structure of poly(tetrafluoroethylene) homo- and copolymers in the high pressure phase [26]. In this case the experimental conditions needed to obtain intensity data successfully were extremely difficult to achieve. For example, an unusually large sample volume of polymer was needed to get measurable diffraction intensities. This required the use of large diamond anvils (1 carat gems) with 1 mm (edge-to-opposite edge) octagonal flats. To prevent the sample, in the form of a rod 0.2 mm long and 0.36 mm in diameter, from being crushed, a special high yield-strength gasket material, Vascomax 300^1^, capable of reaching 5 GPa while still retaining sufficient thickness to prevent pinching the specimen, was used. The gasket diameter was 0.3 mm with a thickness of 0.56 mm. To provide a hydrostatic environment to 5 GPa, a 4:1 methanol:ethanol mixture was used. Under these critical experimental conditions, it was possible to obtain intensity data successfully from a very low-scattering polymer material. The single crystal bond method was employed to measure anisotropic compressibilities of Si and *α*-Pb(N_3_)_2_ [27]. The method provided high sensitivity in determining peak positions and eliminated the effect of centering errors on measured values of 2*θ*. Under optimum conditions diffraction angles were measured with an accuracy of ±0.001° in 2*θ*.

In 1978, NBS underwent a restructuring of its organization in favor of a Laboratory concept and the Section no longer appeared in the organizational charts. Laboratories consisted of Divisions and the former Crystallography Section became part of the Ceramics, Glass, and Solid State Science Division, part of which later became the Ceramics Division. Because of the existence of a pressing National need to help U.S. industry, priorities changed toward new directions because of substantial new responsibilities added by the U.S. Congress to the NBS mission. To help meet these needs the activity of the high pressure group turned to utilizing the DAC to fabricate new dense ceramic materials from nanosize ceramic powders. That activity was ongoing to 1995 and resulted in many advances.

## 6. Conclusions

The invention of the diamond anvil cell (DAC) and the ruby technique were, truly, outstanding achievements of the Crystallography Section. Both had a lasting impact on the advancement of high pressure research in the latter part of the 20th century. The DAC is a unique instrument, unsurpassed in its simple design and mode of operation. It provides the means to generate ultrahigh pressures in a small, portable, simple mechanical device, relatively inexpensive to manufacture and small enough to fit in the palm of one’s hand. Also, it is readily adaptable to a variety of scientific measurement techniques. Because it features a diamond window with 180° transmission, it is particularly suited for x-ray diffraction, spectroscopy, and optical polarizing microscopy. In x-ray crystallography, the DAC was used at NBS, for the first time, for powder diffraction in 1960, and later, after the introduction of the gasket technique, for single crystal diffraction in 1964. Both were benchmark events in the early development of high pressure x-ray crystallography.

Even with its great successes in high pressure x-ray crystallography, the DAC had a serious deficiency—the lack of a rapid, convenient, and reliable method to measure sample pressure. This fault hindered its acceptance as a world-class scientific instrument. The invention of the ruby fluorescence technique in 1971 changed this view dramatically and the device began to be increasingly used world-wide. In 1974, an improved NBS design of the DAC attained pressures (assuming a linear ruby shift) in the 50 GPa range for the first time. The ultimate pressure capability of the DAC had not been established, but the potential for achieving even higher pressures by further improvements in design was recognized. Transition pressures in several semiconductors used as markers in the revised 1970 fixed-point scale were measured by the ruby technique. The results indicated that the generally accepted and widely used fixed-point scale diverged from the ruby scale above 13.5 GPa and disagreed by as much as a factor of two at 50 GPa with the ruby scale defining the lower pressure. As a result of these studies, the fixed-point scale was revised downward, and the ruby scale, widely used to measure pressure in the DAC, became the *de facto* secondary pressure standard. Moreover, it was soon determined that the pressure-shift was essentially independent of temperature within the uncertainty of the measurement at that time, so pressures could be measured at temperatures other than RT. Particularly useful were studies at elevated temperatures where many high pressure physical phenomena occur. There were significant “spin offs” derived from the ruby technique. For x-ray crystallography, an important one, particularly for single crystal work, is the facility to determine the nature of stresses in a pressure-transmitting medium from both line-shift and line-broadening measurements. As early as 1973, such measurements led to the discovery of several pressure-transmitting media that extended the hydrostatic limit to unrivaled pressures for that time. Indeed, all of these advances were milestone events with NBS playing a pivotal role, for, combined, they ignited and fueled an explosion of activity, not only in crystallography, but in many areas of high pressure research. In a relatively short time, the DAC’s versatility in applications to high pressure studies was shown to be unequaled. The worldwide acceptance of the device spurred laboratories in the U.S. and abroad towards the further development of the full potential of this remarkable instrument. That activity is still going on unabated today. Its been estimated conservatively that the number of DAC instruments in use worldwide during the last 40 years is approximately 5000 [28].

## Figures and Tables

**Table 1:** Observed and calculated *d* spacings for iceVI at 0.9 GPa and RT [8]

*d*- spacing (Å)		*hkl*
Obs.	Calc.	
4.3	4.3	111*^a^
3.6	3.6	012
3.4	3.5	210
3.12	3.08	020
2.91	2.93	021*
2.75	2.75	121
2.63	2.67	301
2.51	2.51	022
2.43	2.42	203
2.21	2.22	004*
2.10	2.09	400
2.01	2.00	031*
1.97	1.97	204
1.85	1.85	230
1.75	1.76	124
1.64	1.64	205
1.56	1.57	502*
1.48	1.48	006
1.44	1.44	142*
1.41	1.42	513
1.37	1.37	424
1.34	1.34	035
1.30	1.30	612
1.26	1.26	107
1.21	1.21	151
1.14	1.14	712
1.104	1.104	108
1.084	1.081	615*
1.050	1.049	535*
1.010	1.009	642

a Those indices marked with an asterisk represent multiple lines and only one possible index is given. These data represent the first ever obtained *in situ* from a single crystal under high pressure.

**Table 2:** A comparison of crystal data for benzene I and II [12]

Benzene I (21 °C, 0.07 GPa)	Benzene II (21 °C, 2.5 GPa)
*a* = 7.17	*a* = 5.417 (5) Å^a^
*b* = 9.28	*b* = 5.376 (19)
*c* = 6.65	*c* = 7.532 (7)
	*β* = 110.00 (8)°
Space group *Pbca*	Space group *P*2_1_/*c*
*Z* = 4	*Z* = 2
*ρ*_c_= 1.18 g cm^−3^	*ρ*_c_ = 1.258 g cm^−3^

a The numbers in parentheses are standard deviations on the reported values in units of the last place, obtained from least-squares refinement from 20 experimental values of 2*θ*. The increase in density (*ρ*_c_) for benzene II illustrates the effect of 2.5 GPa.

**Table 3:** Observed and calculated structure factors for benzene II [12]

*h*	*k*	*l*	*F*_obs_	*F*_calc_
1	0	0	26.43	22.55
2	0	0	13.47	14.58
0	0	2	20.53	18.94
1	0	2	12.86	13.54
2	0	2	9.60	13.19
1	0	−2	33.39	33.32
2	0	−2 (L)	5.15	2.62
3	0	−2 (L)	6.98	4.99
0	0	4	6.26	6.73
1	1	0 (L)	3.98	4.20
3	1	0	7.87	8.57
0	1	1	24.37	25.12
1	1	1 (L)	4.98	7.02
2	1	1	10.41	7.54
1	1	−1	16.81	17.05
2	1	−1	11.69	11.29
0	1	2	17.42	18.50
1	1	2 (L)	5.98	1.05
2	1	2 (L)	6.98	2.24
1	1	−2	27.35	27.26
2	1	−2	12.58	11.06
1	1	−3	12.30	12.85
3	1	−3	8.18	8.29
0	1	−4 (L)	6.98	1.42
1	1	−4	11.85	13.60
2	1	−4	11.05	10.43
0	2	0 *	7.35	7.77
0	4	0 *	5.29	5.95

a Reflections marked by an “L” were unobserved because their intensity did not exceed the background. They were assigned an intensity slightly less than the smallest observed value. Those marked by an asterisk were measured on a second crystal which was in a different orientation in the cell. These data represent the first ever obtained *in situ* from a high pressure polymorph of a completely unknown structure.

**Table 4:** Original unit cell and space group data for some high pressure polymorphs obtained from single crystal x-ray diffraction at high pressure and RT [13]

Substance	Crystal system	Unit cell dimensions, Å^a^	Space group
C_6_H_6_–I	orthorhombic	*a* = 7.17, *b* = 9.28, *c* = 6.65	*Pbca*
CS_2_	orthorhombic	*a* = 6.16, *b* = 5.38, *c* = 8.53	*Cmca*
Br_2_	orthorhombic	*a* = 8.54, *b* = 6.75, *c* = 8.63	*Cmca*
CCl_4_ I	rhombohedral	*a* = 14.27, *α* = 90°	
CCl_4_ II	monoclinic	*a* = 22.10, *b* = 11.05, *c* = 25.0, *β* = 114.0°	*Cc* or *C*2/*c*
CCl_4_ III	orthorhombic	*a* = 11.16, *b* = 14.32, *c* = 5.74	*C*222_1_
KNO_3_ III	rhombohedral	*a* = 14.31, *03B1* = 78 54′	
KNO_3_ IV(?)	orthorhombic	*a* = 5.58, *b* = 7.52, *c* = 6.58	*P*2_1_*nb* or *Pmnb*

a All unit cell dimensions are given in Å with estimated uncertainties of ±2 in the last decimal place given and uncertainties of ±5° in angles.

**Table 5:** Lattice parameters of some inorganic azides obtained at various pressures by single crystal x-ray diffraction at RT [14]

Substance	Crystal system	Pressure (bar)	*a* (10^−8^ cm)	*b* (10^−8^ cm)	*c* (10^−8^ cm)	*β* (deg)	*V* (10^−24^ cm^3^)
Pb(N_3_)_2_	Orthorhombic	1	11.31	16.25	6.63		1218.0
		10 395	11.08(5) a	16.16(3)	6.630(5)		1187.0(5)
		22 210	10.83(1)	16.14(1)	6.601(1)		1154.0(2)
Pb(N_3_)_2_	Monoclinic	1	18.46(8)	8.909(8)	5.093(6)	106.2(2)	804.4(4)
		10 395	18.01(9)	8.774(8)	5.065(5)	105.9(2)	770.0(4)
Ba(N_3_)_2_	Monoclinic	1	5.435(4)	4.401(1)	9.611(4)	99.67(8)	226.2(2)
		10 395	5.395(4)	4.345(3)	9.553(6)	99.8(2)	220.2(3)
		22 210	5.375(9)	4.316(3)	9.47(2)	101.2(5)	215.4(6)
KN_3_	Tetragonal	1	6.072		7.144		263.4
		10 395	6.034		6.828		248.6
		22 210	5.992		6.638		238.3
TlN_3_	Tetragonal	1	6.196(8)		7.376(7)		283.2(6)
		2 990	6.178(8)		7.316(7)		279.2(6)
NaN_3_	“Monoclinic”^b^	1	6.630(2)	3.640(2)	5.299(2)	111.5(5)	118.9(2)
		10 395	6.098	3.593	5.288	106.0	111.3

a Numbers in parentheses represent deviations in the last significant figure shown resulting from least-squares fitting process. Where standard deviations are not shown either the parameters were obtained from the literature or there was insufficient data to do a meaningful least-squares refinement.

b NaN_3_ assumed monoclinic at 9 bar and the parameters listed derived on that basis.

**Table 6:** Observed and calculated structure factors for CCl_4_ III [15]

*h*	*k*	*l*	*F*_obs_	*F*_calc_
0	1	1	26.575	26.000
0	1	2	18.833	19.072
0	1	3	46.223	42.693
0	1	5	31.326	33.628
0	1	6	25.074	22.580
0	2	4	23.671	22.330
0	2	3	18.115	18.058
0	2	1	25.379	22.896
0	2	2	17.702	21.996
0	3	1	20.073	19.217
0	3	4	21.095	27.875
0	4	1	11.308	6.888
0	1	4	24.031	28.600
0	0	2	33.955	27.809
0	0	4*^a^	15.091	13.549
0	0	6*	33.846	22.551
0	2	0*	83.291	80.627
0	4	0	19.974	23.681
−1	1	3	88.900	95.104
−1	1	1	52.463	53.041
1	1	4	30.050	31.799
1	1	5	28.924	29.708
1	1	7	29.800	27.337
3	3	3	19.032	17.331
2	2	4	14.399	15.029
2	2	6	20.409	17.126
1	1	3	15.151	13.167
1	1	6	15.526	14.143
−2	2	1	17.154	11.715
2	2	3	27.797	32.311
−2	2	4	33.556	37.779
3	3	1	13.773	13.357
3	3	2	7.513	9.314
−3	3	2	24.416	25.312
−2	2	7	11.770	14.422
−3	3	4	14.149	14.452
−3	3	7	11.645	7.326
4	4	2	14.024	9.977
2	1	5	28.285	31.299
−2	1	1	39.141	40.119
−2	1	2	68.795	80.704
−2	1	3	36.280	44.797
−2	1	5	20.399	19.128
−2	1	6	13.978	9.701
2	1	1	18.181	17.012
2	0	2	136.471	133.854
1	1	1	37.950	37.705
3	1	3	31.748	32.344
1	2	1	115.847	114.322
1	3	1	17.797	13.575
2	2	2*	36.732	40.364
3	2	3	39.749	42.664

a The observed structure factors are for the combined sets of data (reduced to 52 unique reflections) and the calculated structure factors are based on the atomic coordinates of the reference structure. Those reflections marked with an asterisk have averaged observed structure factor values which resulted from combining duplicate reflections from the separate groups of data. These data are shown to illustrate the full potential of the method in attempting an analysis of an unknown high pressure polymorph using a relatively high x-ray scattering molecule compared to those materials studied earlier.

**Table 7:** Lattice constants for some tetrahalogens with the SnBr_4_-type structure (space group, *P*2_1_/*c*) [15]

Substance	*a*_1_ Å	*b*_1_ Å	*c*_1_ Å	*β°*	*a*:*b*:*c*
SnBr_4_ (20 °C)	10.59±0.03	7.10±0.02	10.66±0.03	103.6±0.2	1.492:1:1.501
TiCl_4_ (−32 °C)	9.70	6.48	9.75	102.67	1.495:1:1.501
SnCl_4_ (−39 °C)	9.85	6.75	9.98	102.25	1.460:1:1.480
TiBr_4_ (20 °C)	10.17±0.02	7.09±0.01	10.41±0.01	102.0±0.2	1.434:1:1.468
CCl_4_ III (10 kbar)	9.079±0.021	5.764±0.003	9.201±0.003	104.29±0.05	1.575:1:1.596

## Footnotes

1 Certain commercial equipment, instruments, or materials are identified in this paper to foster understanding. Such identification does not imply recommendation or endorsement by the National Institute of Standards and Technology, nor does it imply that the materials or equipment identified are necessarily the best available for the purpose.
